# Therapeutic Immune Reprogramming by Rapamycin Attenuates Plaque Inflammation and Lymphoid Immune Responses in Aged Atherosclerotic Mice

**DOI:** 10.1111/acel.70730

**Published:** 2026-09-23

**Authors:** Jill de Mol, Daphne H. de Korte, Marie A. C. Depuydt, Virginia Smit, Mireia N. A. Bernabé Kleijn, Peter J. van Santbrink, Christoph J. Binder, Florentina Porsch, Frank H. Schaftenaar, Amanda C. Foks

**Affiliations:** ^1^ Division of BioTherapeutics, Leiden Academic Centre for Drug Research Leiden University Leiden the Netherlands; ^2^ Department of Laboratory Medicine Medical University of Vienna Vienna Austria

**Keywords:** aging, atherosclerosis, basic science research, immunology, senescence, vascular disease

## Abstract

Aging is a major risk factor for atherosclerosis and is accompanied by profound changes in the immune system, including effector T cell expansion, senescent cell accumulation, and increased pro‐inflammatory signaling. Rapamycin, a promising rejuvenation therapeutic that inhibits mTORC1 and modulates immune aging, was investigated for its potential to attenuate pro‐atherogenic immune responses in aged atherosclerotic mice. 80‐ to 90‐week‐old male *Ldlr* ^*−/−*^ mice with established atherosclerotic lesions were treated intraperitoneally with rapamycin or control triweekly for 8 weeks. Systemic and plaque immunity was assessed by flow cytometry or single‐cell RNA‐sequencing. Plaque morphology was evaluated by histology. Rapamycin treatment reduced plaque macrophage content (0.73 ± 0.07 × 10^5^ μm^2^) compared to control (ctrl: 0.95 ± 0.09 × 10^5^ μm^2^, *p* < 0.05) and decreased total T cell numbers in vascular and lymphoid tissues. This was accompanied by a shift from effector to central memory phenotypes, particularly within the CD8^+^ compartment, and a relative enrichment of regulatory CD4^+^ T cells (Tregs). Single‐cell transcriptomics of aortic Tregs revealed increased expression of suppressive genes (*Foxp3*, *Tgfb1*) upon rapamycin. Rapamycin also reduced T follicular helper cells, IL‐21 expression, germinal center B cells, plasma cells, and total and oxidation‐specific epitope‐specific immunoglobulin levels. Additionally, age‐associated B cells were diminished in spleen and lymph nodes, reflecting a broader anti‐inflammatory effect on B cell immunity. Finally, aortic leukocytes displayed profoundly reduced senescent enrichment scores. Rapamycin remodels the immune landscape in aged atherosclerotic mice, promoting regulatory lymphocyte subsets and suppressing antigen‐driven and inflammatory immune responses. These findings highlight the potential of immune‐targeted rejuvenation to mitigate age‐related atherogenic inflammation.

## Graphical abstract

Rapamycin exerts multifaceted immunomodulatory effects in age‐induced atherosclerosis, with pronounced inhibitory effects on adaptive immunity. Rapamycin reduced plaque inflammation and macrophage content, diminished pro‐inflammatory T cell responses, lowered age‐associated B cell frequencies, and attenuated senescence‐associated immune signatures, supporting its potential as an immunomodulatory strategy in aged atherosclerosis. Created in BioRender. de Mol, J. (2026). https://BioRender.com/x09xf08.

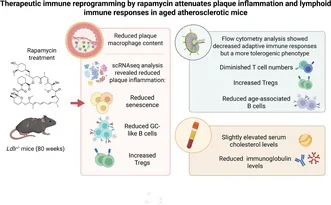

## 1 Introduction

Life expectancy is significantly increasing worldwide and is accompanied by a rising burden of age‐related diseases such as cardiovascular disease (CVD), posing major economical and societal challenges (Zhou et al. 2022). The high prevalence of CVD can partially be explained by increased atherosclerosis progression upon aging (Smit et al. 2023; de Mol et al. 2025). Atherosclerosis is the most prominent underlying pathology of CVD and is characterized by the retention and oxidation of low‐density lipoprotein (LDL) in the vascular intima (Libby 2021). Oxidized LDL (oxLDL) is phagocytosed by macrophages, leading to foam cell formation and plaque development. This process triggers the recruitment of various immune cells from both the innate and adaptive immune system, which can act in either an atheroprotective manner, such as regulatory T cells, B1 cells, and Trem2^hi^ macrophages (Bi et al. 2019), or pro‐atherogenic manner, such as Th1 cells, inflammatory macrophages, and B2 cells (Monaco et al. 2025). In atherosclerosis, the immune balance is disrupted, with a shift towards pro‐inflammatory immunity that drives chronic inflammation and atherosclerosis progression (Rincón‐Arévalo et al. 2020).

Aging contributes to this imbalance through immunosenescence, a functional decline of the immune system (Aiello et al. 2019). Immunosenescence involves skewing of hematopoietic stem cells (HSCs) towards the myeloid lineage, loss of naïve lymphocytes, impaired proliferative capacity (Yang et al. 2021), and accumulation of senescent cells. These senescent cells exhibit a senescence‐associated secretory phenotype (SASP), characterized by persistent cytokine secretion, cell‐cycle arrest, and resistance to apoptosis (Kumari and Jat 2021). The SASP environment fuels chronic low‐grade inflammation, termed inflammaging, that promotes pro‐inflammatory immune responses. The aging immune system is further marked by the emergence of age‐related pro‐inflammatory immune subsets, such as granzyme K‐expressing CD8^+^ T cells and age‐associated B cells (ABCs), which we previously identified in mouse and human atherosclerotic plaques (Smit et al. 2023). This chronic inflammatory shift may render plaques more vulnerable to rupture, potentially increasing the risk of acute cardiovascular events.

It is therefore of utmost importance to explore strategies that target immunosenescence and restore immune balance in atherosclerosis. One such candidate target is rapamycin, an allosteric inhibitor of the mammalian target of rapamycin complex 1 (mTORC1), and to a lesser extent mTORC2 (Sarbassov et al. 2006; Chen et al. 2010). By forming a complex with FK506‐binding protein 12 (FKBP12), rapamycin disrupts mTORC1 function and inhibits mitochondrial RNA translation and cell proliferation (Pearson et al. 1995). In IL‐2 stimulated T cells, rapamycin blocks G1/S phase progression, suppressing effector T cell responses while selectively allowing the expansion of Tregs, which are resistant to mTOR inhibition via phosphatase and tensin homolog (PTEN) and Pim‐2 proto‐oncogene, serine/threonine kinase (PIM2) (Fox et al. 2005). Importantly, mTORC1 activity has been linked to cellular senescence (Saoudaoui et al. 2021). Rapamycin downregulates key senescence markers, such as p53, alleviates oxLDL‐induced senescence in vascular smooth muscle cells (Luo et al. 2017), and rejuvenates aged HSCs both in vivo and ex vivo (Luo et al. 2014; Chen et al. 2009). Furthermore, its capability to inhibit cell proliferation and downregulate p53 has demonstrated efficacy in reducing tumor growth and metastasis, leading to its FDA approval for specific anti‐cancer therapies (Blagosklonny 2023). Preclinical evidence also suggests a beneficial role of rapamycin in other age‐related diseases, including its capability to reduce brain inflammation and lesions (Selvarani et al. 2020).

Further supporting the clinical relevance of rapamycin in atherosclerotic CVD (ASCVD), several studies have shown that rapamycin‐eluting stents in coronary artery disease patients increased Treg numbers (Hester et al. 2012; Potekhina et al. 2011), and mTOR inhibition in young atherosclerotic mice reduced plaque burden by dampening inflammation, chemotaxis, and macrophage infiltration (Castro et al. 2004; Pakala et al. 2005). It is, however, unclear how rapamycin affects age‐associated immune responses in atherosclerosis, which is crucial for the translation of rapamycin‐based therapies to the older CVD population. Due to its dual role in modulating immunity and suppressing senescence, we therefore investigated whether rapamycin‐induced mTOR inhibition could attenuate plaque inflammation and restore immune homeostasis in aged atherosclerotic *Ldlr* ^*−/−*^ mice.

## 2 Methods

### 2.1 Animals

All animal experiments were approved by the Leiden University Animal Ethics Committee and were performed according to the guidelines of the European Parliament Directive 2010/63/EU. Male low‐density lipoprotein receptor‐deficient (*Ldlr* ^*−/−*^) mice on a C57BL/6 background were purchased from Jackson Laboratory (Bar Harbor, Maine, USA) and bred and aged in‐house. All mice were kept under standard laboratory conditions, and diet (Standard Chow: CRM (E), Special Diet Services, Witham, Essex, UK) and water were provided ad libitum. During the experiment, the health status of the mice was assessed weekly by body condition scoring. This non‐invasive method evaluates body condition on a scale from 1 (emaciation) to 5 (obese), based on both visual inspection and palpation, and serves as a key tool for determining humane endpoints when body weight is not a sufficient indicator.

### 2.2 Single‐Cell RNA mTOR Expression

Human carotid endarterectomy plaques were collected from 18 AtheroExpress patients (14 male, 4 female, average age unknown, metadata available in Depuydt et al. 2020). Single‐cell processing and single‐cell RNA sequencing (scRNA‐seq) data analysis were performed as previously described by Depuydt et al. (2020). All studies were performed in accordance with the Declaration of Helsinki and informed consent was obtained from all subjects involved in the study. Single‐cell RNA transcriptomes of CD45^+^ cells from young chow diet‐fed (2‐months‐old, *n* = 9), young Western diet‐fed (containing 0.25% cholesterol and 15% cocoa butter; Special Diet Services, Witham, Essex, UK) (4‐ to 5‐months‐old, *n* = 29), and aged chow diet‐fed (20‐months‐old, *n* = 12) murine aortas of female *Ldlr* ^*−/−*^ mice were retrieved as previously described by Smit et al. (2023).

### 2.3 In Vitro Splenocyte Stimulation

Splenocytes from 80‐week‐old male *Ldlr* ^*−/−*^ mice (*n* = 3) were cultured *in duplo* in RPMI‐1640 completed with 10% FCS, 2 mM L‐Glutamine and 1% Penicillin–Streptomycin. Cells (200,000/well) were stimulated with anti‐CD3 (1 μg/mL, eBioscience, San Diego, USA, 14‐0031‐86) and anti‐CD28 (1 μg/mL, eBioscience, San Diego, USA, 14‐0281‐82) in the absence or presence of 1 or 10 ng/mL rapamycin (Merck, Germany, 553210). After 48 h at 37°C and 5% CO_2_, cells were stained for extracellular markers (Table S1). Flow cytometry was performed on a CytoFLEX S (Beckman Coulter, CA, USA), and data were analyzed using FlowJo v10.8.1 software (Treestar).

For restimulation assays, splenocytes were stimulated for 5 h with phorbol‐12‐myristate 13‐acetate (PMA) (50 ng/mL, Sigma), ionomycin (500 ng/mL, Sigma), and the Golgi‐plug brefeldin A (3 μg/mL, ThermoFisher) to detect intracellular cytokine expression with flow cytometry.

### 2.4 In Vivo Rapamycin Treatment

Male *Ldlr* ^*−/−*^ mice were fed a standard laboratory chow diet for their entire lifespan, allowing gradual development of atherosclerotic lesions. After 80–90 weeks, mice were randomized into a treatment (*n* = 16) or control group (*n* = 16) and administered 1 mg/kg rapamycin (Merck, Germany, 553210) or control solvent (1% DMSO, 5% PEG300, 5% Tween‐80 in phosphate buffered saline [PBS]), respectively, three times a week by intraperitoneal injection. During the experiment, mice were weighed weekly. After 8 weeks of treatment, mice were subcutaneously anesthetized with a cocktail containing ketamine (100 mg/kg) and xylazine (10 mg/kg), and blood was collected via orbital bleeding. Mice were then perfused with PBS through the left cardiac ventricle, after which organs (heart, aortic arch, mediastinal lymph nodes (LN), and spleen) were collected for further analysis. Mice with tumors were excluded from all analyses (*n* = 4; control: *n* = 1, rapamycin: *n* = 3).

### 2.5 Serum Cholesterol, Cytokine and Immunoglobulin Measurements

Blood samples were centrifuged at high speed (8000 rpm) and serum was collected and frozen at −80°C until further use. Total serum cholesterol levels were determined by using an enzymatic colorimetric procedure (Roche/Hitachi, Germany). Precipath standardized serum (Roche/Hitachi) was used as an internal standard. The levels of circulating cytokines were determined using a LEGENDplex (Mouse Th Cytokine 12‐plex, Biolegend, The Netherlands) and measured on a Cytoflex S (Beckman Coulter, USA). Serum IgA, IgM, IgG1, IgG2b, IgG2b, and IgG3 were also determined using a Legendplex (Mouse Immunoglobulin Isotyping 6‐plex, Biolegend, The Netherlands). OSE‐specific antibodies were measured by coating ImmunoGrade 96‐well plates (GmbH) overnight at 4°C with 50 μL/well of MAA conjugated to BSA (MAA‐BSA, 5 μg/mL) or Cu‐oxidized LDL (CuOx‐LDL, 5 μg/mL) diluted in PBS containing 0.27 mM EDTA (pH 7.4). Plates were washed with PBS/EDTA and blocked with 1% BSA in TBS/EDTA (100 μL/well, 1 h, room temperature, RT). After washing, samples diluted as described below in 1% BSA–TBS/EDTA were added in triplicate (3 × 25 μL/well) and incubated overnight at 4°C. For IgM measurements, wells were then incubated for 2 h at RT with alkaline phosphatase–conjugated anti‐mouse IgM (1:20,000 in 1% BSA‐TBS/EDTA; Sigma AP‐9688). For IgG measurements, wells were incubated for 2 h in biotinylated snit‐mouse IgG1 (1:5000, Jackson ImmunoResearch, JIR 115‐065‐205), biotinylated anti‐mouse IgG2b (1:5000, Jackson ImmunoResearch, JIR 115‐065‐207), biotinylated anti‐mouse IgG2c (1:2000, Jackson ImmunoResearch, JIR 115‐065‐208), biotinylated anti‐mouse IgG3 (1:20,000, Jackson ImmunoResearch, JIR 115‐065‐209) diluted in 1% BSA‐TBS/EDTA followed by incubation with alkaline phosphatase‐conjugated NeutrAvidin (1:10,000 in 1% BSA‐TBS/EDTA, Pierce, 31002). All plates were subsequently washed with TBS and developed with 25 μL/well Lumi‐Phos Plus (Lumigen) for 1 h at RT in the dark. Chemiluminescence was recorded on a Synergy2 Gen5 reader (RLU/100 ms). For antigen‐specific isotype measurements, samples were diluted in 1% BSA‐TBS/EDTA as follows: CuOx‐LDL ELISA—IgM 1:1000; IgG, IgG1, IgG3 1:100; IgG2b and IgG2c 1:500. MAA‐BSA ELISA—IgM, IgG, IgG1, IgG3 1:1000; IgG2b and IgG2c 1:5000.

### 2.6 Histology and Immunohistochemistry

Hearts were embedded and frozen in Tissue‐Tek OCT (Sakura Finetek, CA, USA) and stored at −80°C until further use. Cryosections of the aortic root of 10 μm‐thickness were collected with 80 μm distance between sections using a Leica CM1950 Cryostat (Leica Biosystems, Germany). Atherosclerotic lesion size and percentage of occlusion were assessed with Oil‐Red‐O (ORO) and hematoxylin staining in the five largest sections of the aortic root. Three sequential sections, which displayed the highest lesion content, were used for plaque composition. Collagen content was determined using Masson's trichrome staining (Sigma‐Aldrich, USA). Necrotic core size was assessed by manual selection of acellular, debris‐rich regions in the Masson's trichrome stained sections. Corresponding sections on separate slides were stained for monocyte/macrophage content with a MOMA‐2 antibody (1:1000, AbD Serotec) followed by secondary anti‐rat antibody (1:200, AbD Serotec). Secondary antibodies were detected using the Vectastain ABC kit (Vector) and visualized using ImPACT NovaRED substrate (Vector Laboratories, CA, USA). Slides were digitalized with a Panoramic 250 Flash III slide scanner (3DHISTECH, Hungary) or Leica DM‐RE microscope and LeicaQwin software (Leica Imaging Systems, Germany). Stained sections were manually analyzed with ImageJ software. Analysis was performed blinded.

### 2.7 Flow Cytometry

Single‐cell suspensions of lymph nodes and spleens were obtained by mashing organs through a 70 μm cell strainer (Greiner Bio‐One, the Netherlands). Splenocytes were lysed for 2 min with ACK lysis buffer (0.15 M NH_4_Cl, 10 mM NaHCO_3_, 0.1 mM EDTA, pH 7.3) to remove erythrocytes. Aortic arches were digested with 450 U/mL collagenase I, 250 U/mL collagenase XI, 120 U/mL DNAse, and 120 U/mL hyaluronidase for 30 min at 37°C before being mashed through 70 μm cell strainers. Fc block was used to block CD16/CD32 receptors to prevent nonspecific binding (clone 2.4G2, BD Biosciences, CA, USA). Living cells were selected using Fixable Viability Dye eFluor 780 (1:2000, eBioscience), and different cell populations were distinguished with anti‐mouse fluorochrome‐conjugated antibodies. Antibody staining of transcription factors and cytokines was performed using transcription factor fixation/permeabilization concentrate, diluent solutions, and cytofix/permeabilization solutions, respectively (BD Biosciences). Flow cytometry was performed on a CytoFLEX S (Beckman Coulter, CA, USA), and data were analyzed using FlowJo v10.8.1 software (Treestar). All anti‐mouse fluorochrome‐conjugated antibodies used are listed in Table S1.

### 2.8 Real‐Time Quantitative PCR

RNA was extracted from isolated splenocytes using the guanidinium thiocyanate‐phenol chloroform method as described by Chomczynski and Sacchi. cDNA was generated using Maxima H Minus Reverse Transcriptase (200 U/μL, ThermoFisher). Quantitative gene expression analysis was performed using RealQ SYBR Green Master Mix on a QuantStudio 6 Flex Real‐Time PCR (PCR program: 15 min 95°C [25 s 95°C 40 s 62°C] 40×, Applied Biosystems). Gene expression of genes of interest (Table S2) was normalized to the average of three tested housekeeping genes (36B4, peptidyl‐prolyl cis‐trans isomerase, and actin‐beta).

### 2.9 Aortic CD45 Cell Isolation for Single‐Cell RNA‐Sequencing

Atherosclerotic aortic arches, from which perivascular adipose tissue was removed, were isolated from control‐ (*n* = 19) and rapamycin‐treated (*n* = 20) male *Ldlr* ^*−/−*^ mice (75–95 weeks) and enzymatically digested as described earlier. Single‐cell suspensions were stained with Fixable Viability Dye eFluor 780 (1:2000, eBioscience) and CD45‐PE (1:500, clone 30‐F11, Biolegend). After removing doublets, alive CD45^+^ cells were sorted (Figure S1A) in PBS supplemented with 0.04% BSA with a 100 μm nozzle using a FACS Aria II SORP (BD Biosciences) and loaded on a Chromium Single Cell instrument (10× Genomics) to generate single cell bead emulsions. Single‐cell RNA‐sequencing libraries were arranged using the Single‐cell 3′ solution v2 reagent kit (10× Genomics). Sequencing was performed on an Illumina HiSeq2500, and the digital expression matrix was generated by de‐multiplexing barcode processing and gene UMI (unique molecular index) counting using the Cell Ranger v3.0 pipeline (10× Genomics). Data quality is provided in Figure S1B–D.

### 2.10 Single‐Cell Data Processing and Integrative Analysis

Digital expression matrices were analyzed using the Seurat package in R. Single‐cell transcriptomics of CD45^+^ cells from control‐ and rapamycin‐treated *Ldlr* ^*−/−*^ mice were loaded and filtered from doublets and low‐quality cells. Low‐quality cells were removed by setting thresholds for mitochondrial gene expression (maximum of 5%) and unique gene count reads (between 200 and 5500). Thereafter, data were log‐normalized, and gene expression was scaled. Using the DoubletDecon approach, doublets were identified and excluded from the analyses. Transcriptomes of the two datasets were then integrated to perform comparative analysis on the remaining 32,859 cells (ctrl: 15,947 and rapa: 16,912). 50 Principal component analysis (PCA) components were included for clustering at a resolution of 2 and 30 dimensions. Data were visualized through Uniform Manifold Approximation and Projection (UMAP), and the Seurat function FindAllMarkers was used to detect the differentially expressed genes to define the cell clusters. For re‐clustering, myeloid clusters, *Cd79b* ^*+*^ B cell clusters, and *Cd3e* T cell clusters were selected from the main clustering. Myeloid cells that co‐expressed B and T cell markers, likely reflecting macrophages that had efferocytosed lymphocytes, were excluded from subsequent analysis due to their ambiguity in origin. This resulted in a myeloid subclustering (5937 cells) with 19 dimensions and a resolution of 1, a B cell subclustering (10,955 cells) with 10 dimensions and a resolution of 0.5, and a T cell subclustering (8931 cells) with 14 dimensions and a resolution of 1. UMAPs, dot plots, violin plots, volcano plots, and heatmaps were generated in R. Pathway analyses were performed using the pathfindR package in R, using the BIOCARTA gene set as reference.

### 2.11 Statistical Analysis

Data are expressed as mean ± SEM, and outliers were identified and removed with a Grubbs outlier test (*α* = 0.05). Significance of mouse data with two groups was tested using a two‐tailed unpaired *t*‐test or non‐parametric Mann–Whitney *U* test. Significance of stimulated T cells with either control or rapamycin was tested by an ordinary one‐way analysis of variance (ANOVA) test. *p* values of < 0.05 were considered significant. Statistical analysis was performed using GraphPad Prism 9.0.

## 3 Results

### 3.1 mTOR Is Expressed in Human and Mouse Atherosclerotic Plaques

**FIGURE 1:**
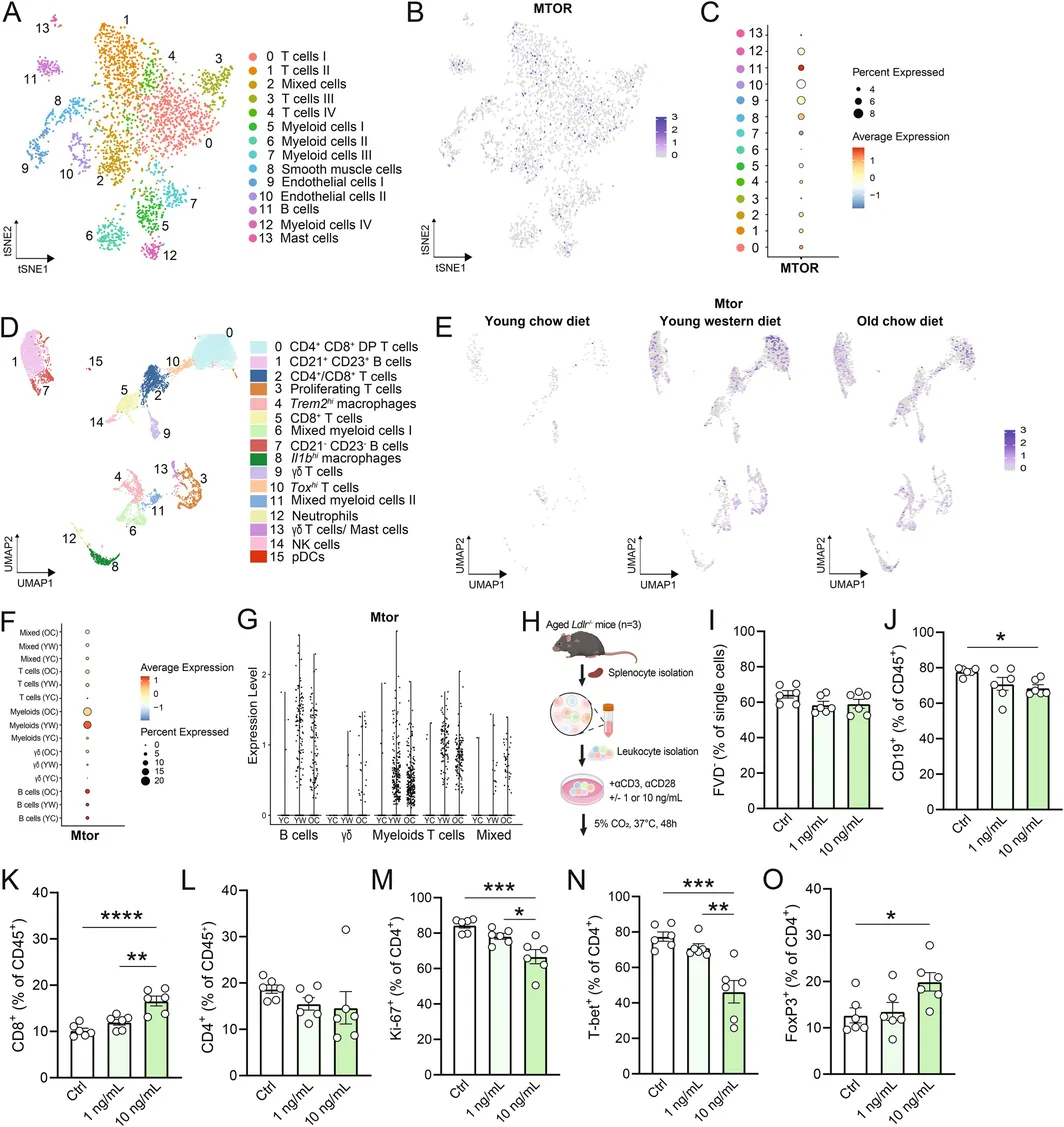
*Mtor* expression in human and mouse atherosclerotic plaques. (A) Single‐cell RNA sequencing of human carotid artery plaques identified 14 distinct cell clusters, as produced in (Depuydt et al. 2020). (B) FeaturePlot and (C) DotPlot visualization of *MTOR* expression across human carotid artery clusters of 18 patients, showing highest expression in B cell cluster (11). (D) scRNAseq of aortic arch leukocytes of young chow diet (YC)‐fed (2‐months‐old, *n* = 9), young Western diet (YW)‐fed (4‐ to 5‐months‐old, *n* = 29) and old chow diet (OC)‐fed (20‐months‐old, *n* = 12) female *Ldlr* ^*−/−*^ mice resulted in 16 distinct cell clusters (Smit et al. 2023). (E) FeaturePlot visualization of *Mtor* expression across murine aortic arch clusters. (F) DotPlot and (G) ViolinPlot visualization of *Mtor* expression in murine aortic arch B cells, γδ T cells, myeloids, T cells and mixed immune cells, split by condition. (H) Experimental setup of in vitro αCD3/αCD28 splenocyte culture in the absence or presence of 1 or 10 ng/mL rapamycin. The percentage of (I) Fixable Viability Dye (FVD)^−^ live cells, (J) CD19^+^ B cells, (K) CD8^+^ T cells, (L) CD4^+^ T cells, (M) proliferating Ki‐67^+^ CD4^+^ T cells, (N) T‐bet^+^ CD4^+^ T cells, and (O) FoxP3^+^ CD4^+^ T cells were measured using flow cytometry. In vitro data are from *n* = 3 mice per group. Mean ± SEM plotted. Statistical significance was tested using a one‐way ANOVA. *p < 0.05, **p < 0.01, ***p < 0.001, ****p < 0.0001. Created in BioRender. de Mol, J. (2026). https://BioRender.com/l2oe89g.

We first aimed to determine the expression of *MTOR*, the gene encoding for the mTOR protein, which is part of both mTORC1 and mTORC2, in human and mouse atherosclerosis. Although the potential role of mTOR signaling in cardiovascular pathology has been highlighted (Sciarretta et al. 2018), its cellular expression profile in human atherosclerotic plaques has not yet been characterized. Previously, our group employed scRNA‐seq to profile the cellular transcriptome of human carotid artery plaques from 18 patients (Depuydt et al. 2020), which resulted in 14 distinct cell clusters, including three non‐immune cell types and eleven leukocyte clusters comprising myeloid cells, T cells, B cells, and mast cells (Figure 1A). Notably, *MTOR* was broadly expressed across these clusters (Figure 1B), with B cells among the cell types showing relatively high expression (Figure 1C). To gain more insights into the influence of aging and atherosclerosis on *MTOR* expression, we analyzed our previously published murine scRNA‐seq dataset, comparing aortic tissue from young chow diet‐fed (YC) (Cochain et al. 2018), young Western‐diet fed (YW), and old chow‐diet fed *Ldlr* ^*−/−*^ (OC) mice (Figure 1D) (Smit et al. 2023). Both aging and a Western‐diet elevated *Mtor* levels in T cells and macrophages (Figure 1E–G), whereas B cells displayed the strongest average expression (Figure 1F,G). Given the prominent *Mtor* expression in adaptive immune cells, we next assessed their sensitivity to mTOR inhibition in vitro. Splenocytes from aged *Ldlr* ^*−/−*^ mice (*n* = 3) were cultured for 48 h in the presence or absence of 1 or 10 ng/mL rapamycin (Figure 1H). While neither concentration compromised overall cell viability (Figure 1I), exposure to 10 ng/mL significantly reduced total B cell frequency (ctrl: 77.87% ± 0.89%, vs. 10 ng/mL: 68.66% ± 1.78%, *p* < 0.05) (Figure 1J), which was accompanied by a relative increase in CD8^+^ T cells (Figure 1K). Treatment with rapamycin for 48 h significantly impaired CD4^+^ T cell proliferation (ctrl: 84.47% ± 1.60%, vs. 10 ng/mL: 66.70% ± 4.00%, *p* < 0.001), despite stable overall frequencies (Figure 1L,M). This was accompanied by a shift towards a more tolerogenic phenotype, with fewer T‐bet^+^ (ctrl: 77.42% ± 2.59%, vs. 10 ng/mL: 46.28% ± 6.36%, *p* < 0.05) and more FoxP3^+^ CD4^+^ T cells (ctrl: 12.71% ± 1.61%, vs. 10 ng/mL: 19.93% ± 1.99%, *p* < 0.05) (Figure 1N,O). Together, these results show that *Mtor* is highly expressed in adaptive immune cells in atherosclerotic plaques and that its inhibition in vitro alters both their abundance and functional phenotype.

### 3.2 Rapamycin Modulates Immune Cell Populations Without Affecting Plaque Morphology

**FIGURE 2:**
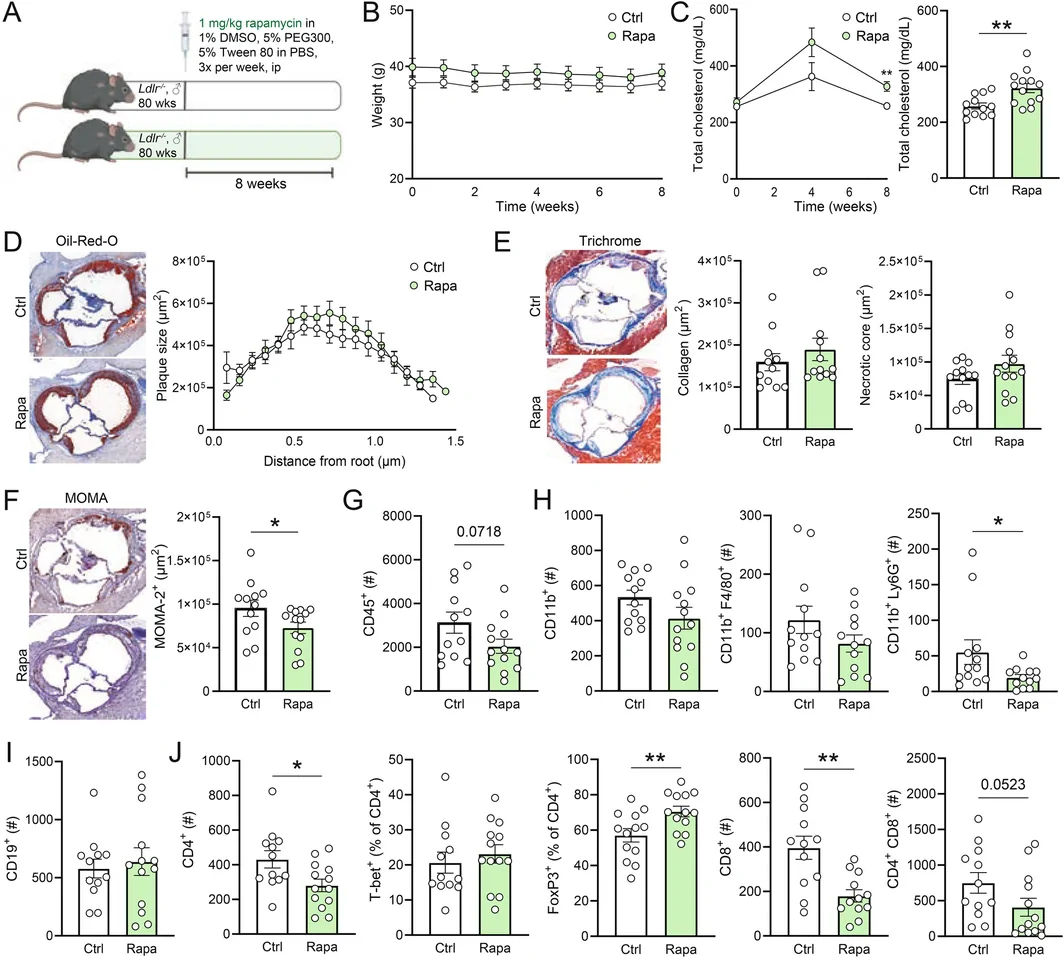
Rapamycin attenuates plaque inflammation. (A) Experimental setup: Male *Ldlr* ^*−/−*^ mice aged 80 weeks were administered a 1 mg/kg rapamycin (rapa; green bars) or control solvent (1% DMSO, 5% PEG300, 5% Tween80 in PBS; ctrl; white bars), 3 times a week intraperitoneally (ip), for 8 weeks. (B) Body weights and (C) serum cholesterol levels were measured during the experiment. (D) Lesion size and volume in the aortic root was determined using an Oil‐Red‐O and hematoxylin staining. (E) Collagen content was determined using a Masson's trichrome staining and (F) macrophage/monocyte content was assessed with a MOMA‐2 antibody. Representative pictures are shown with a magnitude of 5X. Flow cytometry analysis was performed to show differences in (G) total aortic leukocytes; (H) CD11b^+^ myeloid cells, including F4/80^+^ macrophages and Ly6G^+^ neutrophils; (I) CD19^+^ B cells and; (J) T cells, including CD4^+^, Th1 (T‐bet^+^), Tregs (FoxP3^+^), CD8^+^ and double‐positive CD4^+^ CD8^+^ T cells. All data are from *n* = 12 mice per group. Mean ± SEM plotted. Statistical significance was tested using a *t*‐test. *p < 0.05, **p < 0.01. Created in BioRender. de Mol, J. (2026). https://BioRender.com/8uzkqxn.

Building on these in vitro findings, we next investigated the impact of rapamycin‐mediated mTOR inhibition in vivo using aged atherosclerotic mice. 80‐ to 90‐week‐old male *Ldlr* ^*−/−*^ mice on a chow diet were treated intraperitoneally three times a week with either rapamycin (*n* = 16) or a control solvent (*n* = 16) for 8 weeks (Figure 2A). Rapamycin treatment did not affect body weight (Figure 2B), but significantly increased total cholesterol levels compared to controls (*t* = 8, ctrl: 258.4 ± 10.7 mg/dL vs. rapa: 322.7 ± 16.3 mg/dL, *p* < 0.01; Figure 2C), which is in line with previously reported effects of rapamycin (Gadioli et al. 2009; Kasiske et al. 2008). Despite increased cholesterol levels, histological analyses showed that overall plaque size and volume (Figure 2D), collagen content, and necrotic core area (Figure 2E) were not affected by rapamycin treatment. We did observe a significant reduction in plaque macrophage content in rapamycin‐treated mice, at both absolute level (ctrl: 0.95 ± 0.09 × 10^5^ μm^2^ vs. rapa: 0.73 ± 0.07 × 10^5^ μm^2^, *p* < 0.05; Figure 2F) as well as percentage of plaque area. To further characterize the effects of rapamycin on plaque inflammation, we next assessed CD45^+^ immune cell counts in the aortic arch using flow cytometry. We observed a trend towards reduced total CD45^+^ cells in rapamycin‐treated mice (ctrl: 3127 ± 482 vs. rapa: 2046 ± 324, *p* = 0.0718; Figure 2G). More detailed analysis showed a significant decrease in CD11b^+^ Ly6G^+^ neutrophils (ctrl: 55 ± 17 vs. rapa: 20 ± 4, *p* < 0.05) in rapamycin‐treated mice, while CD11b^+^ F4/80^+^ macrophage and CD19^+^ B cell numbers remained unchanged (Figure 2H,I). Moreover, rapamycin treatment reduced the total number of CD4^+^ T cells. Within the CD4^+^ T cell compartment, the relative frequency of atheroprotective Tregs was increased (ctrl: 57.08% ± 3.77% vs. rapa: 70.6% ± 3.00%, *p* < 0.01), whereas pro‐inflammatory Th1 cells were unaltered (Figure 2J). Additionally, rapamycin reduced the numbers of both CD8^+^ T cells (ctrl: 396 ± 52 vs. rapa: 180 ± 28, *p* < 0.05) and double‐positive CD4^+^ CD8^+^ T cells (ctrl: 750 ± 144 vs. rapa: 408 ± 125, *p* = 0.052). The relative distribution of major immune cell subsets remained largely unchanged (Figure S2). Collectively, these results indicate that rapamycin alters the inflammatory cell profile within plaques, without affecting overall plaque morphology.

### 3.3 Single‐Cell RNA‐Sequencing Reveals Cell Type‐Specific Immunomodulation by Rapamycin

**FIGURE 3:**
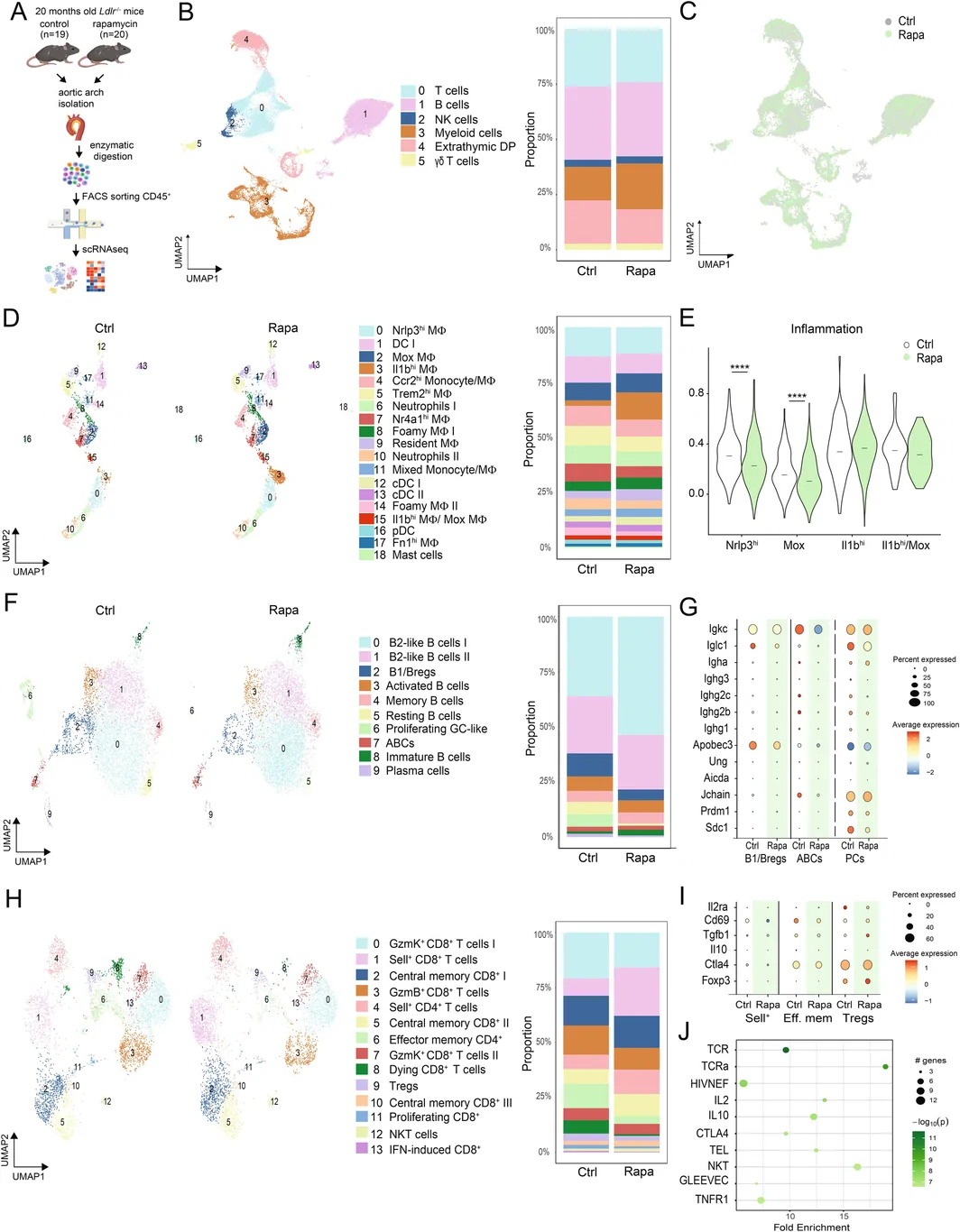
Single‐cell RNA‐sequencing reveals rapamycin‐induced leukocyte alterations in atherosclerotic aortas. (A) Workflow of single‐cell RNA‐sequencing on aortic CD45^+^ cells of control‐ (*n* = 19) and rapamycin‐treated (*n* = 20) aged male *Ldlr* ^*−/−*^ mice. UMAP visualization of clustered aortic leukocytes split by (B) main immune cell clusters, with their relative distribution in a stacked diagram or by (C) group. (D) UMAP plots and stacked diagram visualizing the identified myeloid subclusters. (E) Violin plot showing the inflammation score (Inflammation gene set) for the main pro‐inflammatory macrophage subsets. (F) UMAP plots and stacked diagram visualizing the identified B cell subclusters. (G) Dot plot showing the average expression of genes involved in plasma cell differentiation for B1/Bregs, age‐associated B cells (ABCs) and plasma cells (PCs). The PC cluster was analyzed separately. (H) UMAP plots and stacked diagram visualizing the identified T cell subclusters. (I) Dot plot showing the average gene expression of regulatory T cell (Treg) genes for naïve CD4^+^ T cells, effector memory CD4^+^ T cells, and Tregs. (J) Plot showing the top 10 enriched pathways in rapamycin‐treated T cells as analyzed with pathfindR. Mean ± SEM plotted. Statistical significance was tested using Kruskal–Wallis test. ****p < 0.0001. Created in BioRender. ABC, age‐associated B cell; cDC, conventional dendritic cell; DP, double‐positive; MФ, macrophage; NK, natural killer; pDC, plasmacytoid dendritic cell; Tregs, regulatory T cells.

To gain deeper insights in the immune landscape within atherosclerotic plaques upon rapamycin treatment, we performed single‐cell RNA‐sequencing on cells isolated from the aortic arches of control‐ (*n* = 19) and rapamycin‐treated (*n* = 20) 80‐ to 90‐week‐old male *Ldlr* ^*−/−*^ mice (Figure 3A). Unsupervised clustering identified 45 subclusters (Figure S3A, Table S3), which could be annotated as 6 major immune cell populations: myeloid cells, NK cells, B cells, plasma cells, conventional T cells, γδ T cells, and extrathymic double‐positive (CD4^+^ CD8^+^) T cells based on canonical marker genes (Figure 3B,C, Figure S3B). Overall, rapamycin treatment reshaped the immune landscape, with a relative reduction in extrathymic double‐positive T cells and an increase in myeloid cells.

Myeloid subclustering resolved 19 distinct populations (Figure 3D), including dendritic cells (DCs), monocytes, neutrophils, and various macrophage subsets (Figure S3C, Table S4). Rapamycin altered macrophage composition, increasing *Il1b* ^*hi*^ inflammatory macrophages while reducing *Nlrp3* ^*hi*^ and *Ccr2* ^*hi*^ subsets, and decreasing *Trem2* ^*hi*^ macrophages, whereas foam‐like macrophages were slightly increased. In line with our flow cytometry data, neutrophil clusters were consistently reduced, while dendritic cells and mast cells were not affected. Although subset‐specific variation was observed, inflammatory gene signatures (Table S5) within *Nlrp3* ^*hi*^ and *Mox* macrophages were overall reduced in rapamycin‐treated mice (Figure 3E).

B cell and plasma cell subclustering identified 10 different subsets (Figure 3F, Figure S3D,E, Table S6). While most B2‐like (cluster 0 and 1; *Fcer2a*, *Cr2*), activated (cluster 3; *Myc* ^hi^ *Egr3* ^hi^), and memory (cluster 4; *Bach22* ^*+*^, *Cd83* ^*+*^) B cell populations were relatively unchanged, rapamycin reduced resting B cells (cluster 5; ribosomal genes) and B1/Breg‐like cells (cluster 2; *S100a6*, *Ebi3*, *Cd9*). Strikingly, cluster 6, enriched for proliferating germinal center (GC) B cell markers (*Mef2b*, *Aicda*, *Fas*, *Mki67*), suggesting these cells are derived from GCs, were nearly absent following rapamycin treatment, accompanied by a reduction in plasma cells (cluster 9; *Jchain*, *Prdm1*, *Sdc1*, *Xbp1*). Consistently, expression of plasma cell‐ and immunoglobulin‐related genes (e.g., *Jchain*, *Sdc1*, *Iglc1*, and *Ighg2c*) was reduced (Figure 3G), indicating suppression of antigen‐driven humoral responses.

In‐depth analysis of conventional T cells revealed 14 subpopulations (Figure 3H, Table S7), broadly separating into CD4^+^ and CD8^+^ T populations (Figure S3F,G). Rapamycin drastically reduced effector memory CD4^+^ (cluster 6; *Cd44* ^hi^, *Sell* ^*−*^, *Jun* ^low^) and CD8^+^ (clusters 0 and 7; *Gzmk* ^*+*^, *Tox* ^hi^, and cluster 3; *Gzma* ^hi^, *Gzmb* ^hi^) T cell subsets, while increasing naïve (clusters 1 and 4; *Cd44* ^low^, *Sell* ^+^, *Jun* ^+^) and central memory (cluster 5: *Cd44* ^*+*^, *Sell* ^*+*^) subsets. Proliferating (cluster 11; *Mki67*, *Top2a*, *Hells*) and IFN‐induced CD8^+^ T cells (cluster 13; *Ifit1*, *Ifit3*, *Ifi204*) remained largely unaffected, whereas apoptotic/dying CD8^+^ T cells (cluster 8; mitochondrial genes, *Malat1*) were nearly absent after rapamycin treatment. Importantly, Tregs (cluster 9; *Foxp3*, *Tnfrsf4*, *Ctla4*) were preserved and showed elevated expression of *Foxp3* and *Tgfb1* (Figure 3I), together with enrichment of IL‐10 and CTLA‐4 signaling pathways (Figure 3J), indicating enhanced immunoregulatory activity.

Together, these data demonstrate that rapamycin broadly reshapes the immune landscape of atherosclerotic lesions by modulating myeloid cell states, suppressing antigen‐driven B cell responses, and shifting T cell populations towards a more naive and regulatory phenotype.

### 3.4 mTOR Inhibition Dampens Immune Responses in Lymph Nodes While Preserving Regulatory T Cells

**FIGURE 4:**
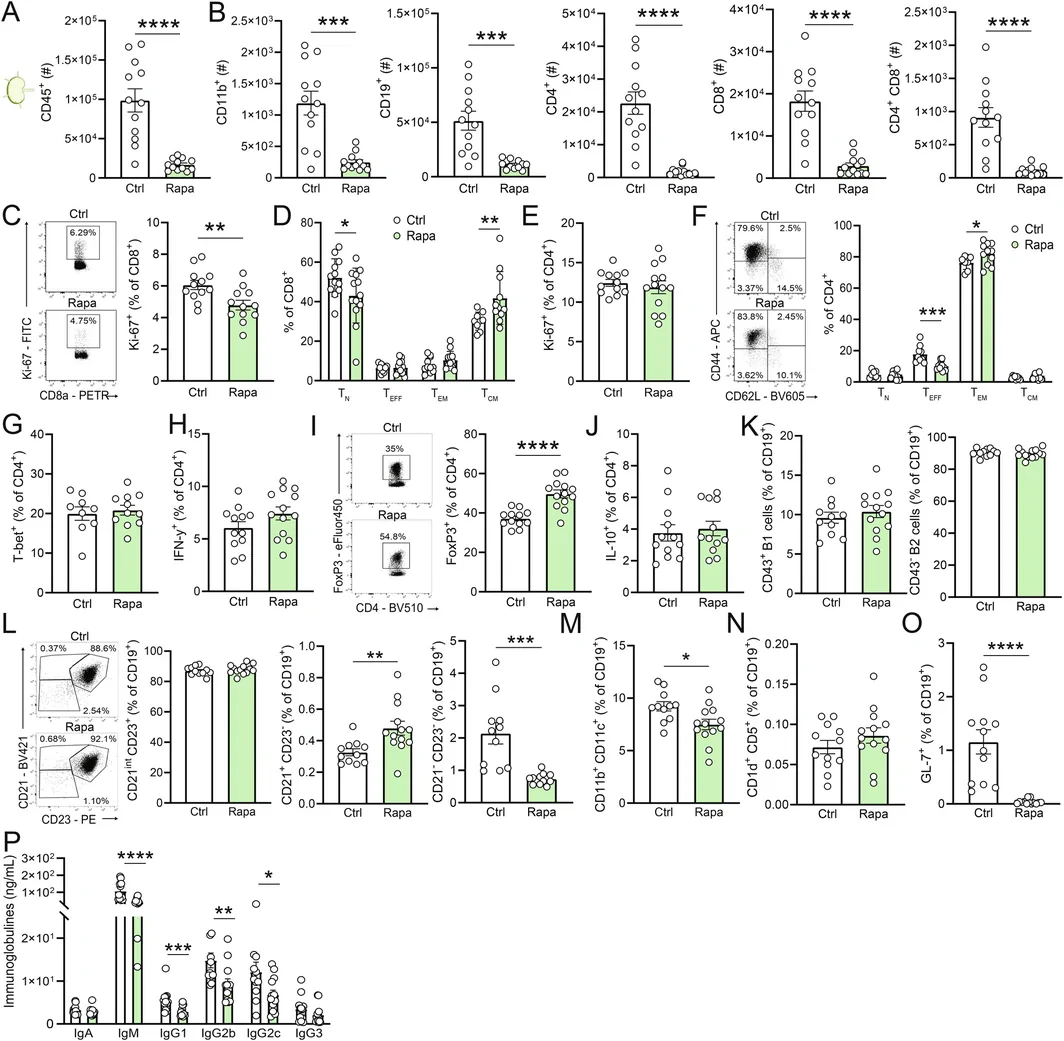
Rapamycin abolishes local inflammation. Flow cytometry analysis of (A) total CD45^+^ immune cell counts in the mediastinal lymph nodes, including (B) CD11b^+^ myeloid cells, CD19^+^ B cells, CD4^+^ T cells, CD8^+^ T cells, and double‐positive CD4^+^ CD8^+^ T cells. (C) Proportion of proliferating (Ki‐67^+^) CD8^+^ T cells and (D) distribution of naïve, effector‐like, effector‐memory and central‐memory phenotype within CD8^+^ T cells. (E) Proportion of proliferating CD4^+^ T cells and its (F) naïve/memory distribution. Frequency of (G) T‐bet^+^ Th1 cells and, upon stimulation, (H) IFNγ‐producing CD4^+^ T cells. In contrast, the frequency of anti‐inflammatory (I) FoxP3^+^ regulatory T cells, and, upon stimulation, (J) IL‐10^+^ frequency were calculated. Flow cytometry was also used to determine the distribution of (K) B1 and B2 cells; (L) CD21^int^ CD23^+^ follicular, CD21^+^ CD23^−^ marginal zone, and CD21^−^ CD23^−^ double negative B cells; (M) CD11b^+^ CD11c^+^ age‐associated B cells; (N) CD1d^+^ CD5^+^ regulatory B cells; and (O) GL‐7^+^ germinal center B cells. A LEGENDplex was performed to measure the (P) level of circulating immunoglobulins. Representative dot plots are shown. Data are from *n* = 12 mice per group. Mean ± SEM plotted. Statistical significance was tested using a *t*‐test. *p < 0.05, **p < 0.01, ***p < 0.001, ****p < 0.0001.

Given the impact of rapamycin treatment on plaque inflammation, we further assessed its effects on the immune landscape in mediastinal lymph nodes (LN) located near the heart and aorta. Strikingly, rapamycin treatment diminished the total immune cell population in the LN by almost 6‐fold (ctrl: 9.9 ± 1.5 × 10^4^ vs. rapa: 1.7 ± 0.2 × 10^4^, *p* < 0.0001; Figure 4A), attributed to an overall decline in myeloid cells, B cells, and T cells (Figure 4B). In line with the markedly reduced T cell pool, rapamycin reduced the percentage of proliferating CD8^+^ Ki‐67^+^ T cells (ctrl: 6.06% ± 0.29% vs. rapa: 4.79% ± 0.31%, *p* < 0.01; Figure 4C). Furthermore, rapamycin treatment resulted in a relative increase in central memory CD8^+^ T cells (ctrl: 30.16% ± 1.52% vs. rapa: 41.85% ± 4.04%, *p* < 0.05; Figure 4D) and reduced the frequency of naïve CD8^+^ T cells (ctrl: 52.15% ± 2.76% vs. rapa: 43.09% ± 3.85%, *p* < 0.05). Within the few remaining CD4^+^ T cells, Ki‐67 expression remained unaltered (Figure 4E), but the relative frequency of effector memory CD4^+^ T cells increased (ctrl: 76.33% ± 1.55% vs. rapa: 81.93% ± 2.08%, *p* < 0.05; Figure 4F) at the cost of effector‐like CD4^+^ T cells (ctrl: 17.79% ± 1.84% vs. rapa: 10.22% ± 0.84%, *p* < 0.001). T‐bet^+^ CD4^+^ T cell percentages, and Th1‐associated IFNγ production upon PMA and ionomycin stimulation, were not affected in rapamycin‐treated mice (Figure 4G,H). Consistent with the aorta, rapamycin induced a robust regulatory CD4^+^ T cell response in the LN (ctrl: 37.18% ± 1.22% vs. rapa: 49.67% ± 2.01%, *p* < 0.0001; Figure 4I), but IL‐10 production was not altered (Figure 4J). In‐depth analysis of the B cell compartment showed no differences in the B1/B2 distribution in the LN (Figure 4K). The majority of B cells consisted of follicular (FO) B2 cells, which remained unaffected upon treatment while the proportion of marginal zone (MZ) B‐like cells slightly increased (ctrl: 0.33% ± 0.02% vs. rapa: 0.48% ± 0.04%, *p* < 0.01; Figure 4L). In contrast, CD23^−^ CD21^−^ B cell frequencies sharply declined (ctrl: 2.14% ± 0.33% vs. rapa: 0.73% ± 0.05%, *p* < 0.001), with a significant decrease in ABCs (ctrl: 9.22% ± 0.44% vs. rapa: 7.50% ± 0.50%, *p* < 0.01; Figure 4M), which comprise most of the CD23^−^ CD21^−^ population, and no effect on regulatory B cells (Figure 4N). Consistent with observations in the aorta, the frequency of GC B cells in the LN was almost diminished (ctrl: 1.16% ± 0.23% vs. rapa: 0.04% ± 0.01%, *p* < 0.0001; Figure 4O), which further coincided with an overall reduction in serum IgM (ctrl: 1.06 ± 0.15 × 10^2^ ng/mL vs. rapa: 4.15 ± 0.05 × 10^1^ ng/mL, *p* < 0.0001) and IgG (IgG1 ctrl: 5.51 ± 0.77 ng/mL vs. rapa: 2.89 ± 0.35 ng/mL, *p* < 0.001; IgG2b ctrl: 1.48 ± 0.17 × 10^1^ ng/mL vs. rapa: 0.93 ± 0.12 × 10^1^ ng/mL, *p* < 0.01; IgG2c ctrl: 1.21 ± 0.23 × 10^1^ ng/mL vs. rapa: 0.68 ± 0.11 × 10^1^ ng/mL, *p* < 0.05) levels (Figure 4P). Additionally, the overall level of antibodies against the oxidation‐specific epitopes (OSE) malondialdehyde (MAA) and oxLDL were also significantly reduced after rapamycin treatment (Figure S4). Altogether, these findings indicate that rapamycin reduces immune cell activation in plaque‐draining lymph nodes while maintaining regulatory T cell populations.

### 3.5 Rapamycin Reshapes Splenic T and B Cell Compartments

**FIGURE 5:**
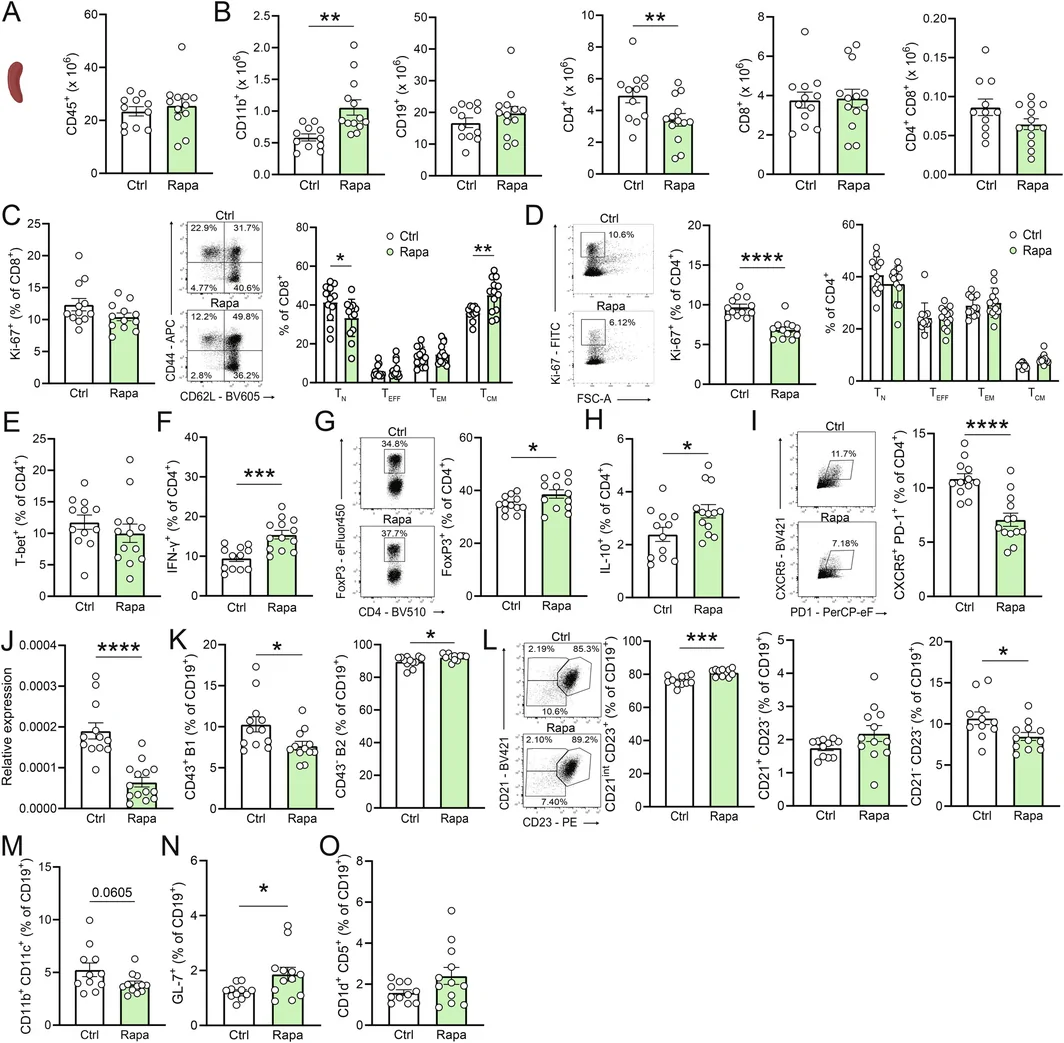
Rapamycin induces a tolerogenic CD4^+^ T cell phenotype systemically. Flow cytometry analysis of (A) total CD45^+^ immune cell counts in the spleen, including (B) CD11b^+^ myeloids, CD19^+^ B cells, CD4^+^ T cells, CD8^+^ T cells, and double‐positive CD4^+^ CD8^+^ T cells. (C) Proportion of proliferating (Ki‐67^+^) CD8^+^ T cells and their distribution of naïve, effector‐like, effector‐memory, and central‐memory phenotype. (D) The proliferation and memory distribution within CD4^+^ T cells was also investigated. Frequency of (E) T‐bet^+^ Th1 cells and, upon stimulation, (F) IFNγ‐producing CD4^+^ T cells and anti‐inflammatory (G) FoxP3^+^ regulatory T cells, and, upon stimulation, (H) IL‐10^+^ CD4^+^ T cells. (I) Flow cytometry analysis of splenic CXCR5^+^ PD‐1^+^ follicular helper T cells. (J) Relative gene expression, as measured with qPCR, of IL‐21 in splenocytes. The proportion of splenic (K) CD43^+^ B1 and CD43^−^ B2 cells; (L) CD21^int^ CD23^+^ follicular, CD21^+^ CD23^−^ marginal zone, and CD21^−^ CD23^−^ B cells; (M) CD11b^+^ CD11c^+^ age‐associated B cells; (N) GL‐7^+^ germinal center B cells; and (O) CD1d^+^ CD5^+^ regulatory B cells was measured using flow cytometry. Representative dot plots are shown. Data are from *n* = 12 mice per group. Mean ± SEM plotted. Statistical significance was tested using a *t*‐test. *p < 0.05, **p < 0.01, ***p < 0.001, ****p < 0.0001.

In contrast to the LN, total immune cell numbers in the spleen were not affected by rapamycin treatment (Figure 5A). However, a similar decrease in splenic CD4^+^ T cells was observed in rapamycin‐treated compared to control mice (ctrl: 4.9 ± 0.47 × 10^6^ vs. rapa: 3.4 ± 0.40 × 10^6^, *p* < 0.05), accompanied by an increase in myeloid cells (ctrl: 0.59 ± 0.05 × 10^6^ vs. rapa: 1.1 ± 0.12 × 10^6^, *p* < 0.01; Figure 5B). Moreover, rapamycin increased the CD8^+^ T cell central memory subset (ctrl: 35.91% ± 1.11% vs. rapa: 45.17% ± 2.36%, *p* < 0.05; Figure 5C) and reduced CD4^+^ T cell proliferation (ctrl: 9.73% ± 0.36% vs. rapa: 6.88% ± 0.34%, *p* < 0.0001; Figure 5D) without affecting the splenic CD4^+^ T cell memory phenotype. Although Th1 frequencies were unchanged (Figure 5E), stimulation of splenocytes revealed an increased proportion of IFNγ‐producing CD4^+^ T cells (ctrl: 9.51% ± 0.82% vs. rapa: 15.43% ± 1.08%, *p* < 0.001; Figure 5F), without corresponding increases in systemic cytokine levels (Figure S5), indicating no systemic inflammatory activation. In line with our findings in plaque and LN, regulatory T cells were increased in the spleen (ctrl: 34.78% ± 0.89% vs. rapa: 38.66% ± 1.60%, *p* < 0.05; Figure 5G), and IL‐10‐producing CD4^+^ T cells were also elevated upon stimulation (ctrl: 2.39% ± 0.27% vs. rapa: 3.27% ± 0.25%, *p* < 0.05; Figure 5H), consistent with a shift towards a more regulatory CD4^+^ T cell profile. Given the importance of follicular helper T cells (Tfh) in B cell activation (Petersone et al. 2023; Snijckers and Foks 2024), we next analyzed this compartment and observed a marked reduction in the frequency of Tfh cells (ctrl: 10.84% ± 0.47% vs. rapa: 7.07% ± 0.61%, *p* < 0.0001; Figure 5I), accompanied by decreased *Il21*gene expression in splenocytes (ctrl: 1.9 ± 0.20 × 10^−4^ RPM vs. rapa: 0.65 ± 0.18 × 10^−4^ RPM, *p* < 0.0001; Figure 5J). In parallel, rapamycin modulated the B cell compartment, increasing the proportion of B2 cells (ctrl: 89.71% ± 0.92% vs. rapa: 92.32% ± 0.55%, *p* < 0.01; Figure 5K). Specifically, we observed elevated FO B cells (ctrl: 76.13% ± 0.87% vs. rapa: 81.11% ± 0.65%, *p* < 0.001; Figure 5L), while MZ B cell proportions remained unaffected. Similar to LN, CD23^−^ CD21^−^ B cell frequencies declined (ctrl: 10.70% ± 0.77% vs. rapa: 8.48% ± 0.49%, *p* < 0.05), with a trend towards decreased ABCs (*p* = 0.0605; Figure 5M). GC B cell frequencies slightly increased following rapamycin treatment (ctrl: 1.22% ± 0.08% vs. rapa: 1.87% ± 0.25%, *p* < 0.05; Figure 5N), while regulatory B cells remained unaffected (Figure 5O). Collectively, these data indicate that rapamycin induces coordinated changes across T and B cell compartments in the spleen, characterized by reduced Tfh cell activity and age‐associated B cell populations, alongside preservation of regulatory immune subsets.

### 3.6 Altered Senescence‐Associated Gene Expression

**FIGURE 6:**
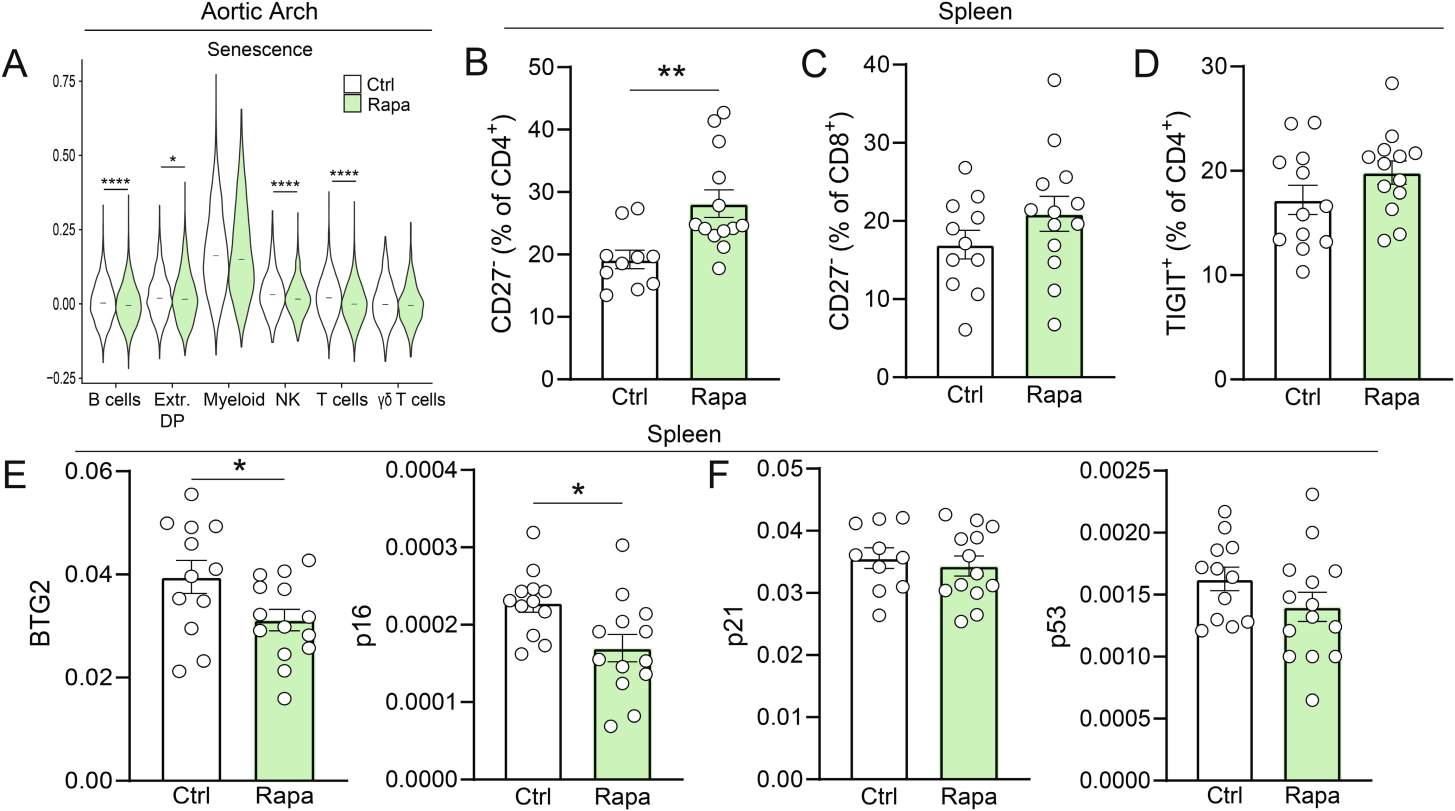
Rapamycin affects senescence‐associated phenotype. (A) Violin plot showing the senescence (SenMayo gene set) (Gomez‐Cambronero 2003) enrichment score of the main aortic immune cell clusters split by treatment. Flow cytometry analysis of CD27 expression in (B) CD4^+^ and (C) CD8^+^ T cells. (D) Frequency of TIGIT^+^ CD4^+^ T cells. Relative gene expression, as measured with qPCR, of (E) BTG2 and p16, and (F) p21 and p53. Data are from *n* = 12 mice per group. Mean ± SEM plotted. Statistical significance was tested using a *t*‐test. *p < 0.05, **p < 0.01.

The potential of rapamycin to target cellular senescence led us to evaluate the SenMayo enrichment score, a gene set marking senescent phenotypes through key proliferation, survival, and SASP‐related genes (Saul et al. 2022) in aortic leukocytes of aged mice (Figure 6A). Among the major immune cell populations, the highest SenMayo enrichment scores were observed in myeloid cells. However, no significant differences were found between control‐ and rapamycin‐treated mice within this cluster. In contrast, the SenMayo enrichment scores in B cells, extrathymic double‐positive T cells, NK cells, and conventional T cells were markedly reduced following rapamycin treatment. Further examination of senescence in the spleen showed an increased proportion of CD27^−^ CD4^+^ T cells upon rapamycin treatment (ctrl: 19.19% ± 1.48% vs. rapa: 28.15% ± 2.21%, *p* < 0.01; Figure 6B). Since CD27 loss is commonly associated with repeated antigen exposure and reduced proliferative potential (Larbi and Fulop 2014), this might suggest a shift towards a more differentiated or senescent phenotype (Chen et al. 2023). In contrast, CD27 expression on splenic CD8^+^ T cells, and expression of the co‐inhibitory receptor TIGIT, another marker implicated in CD4^+^ T cell exhaustion and senescence (van der List et al. 2021), remained unaffected (Figure 6C,D). To evaluate broader senescence‐associated gene expression, we performed qPCR on total splenocytes. Expression of *Btg2* and *Cdkn2a* (encoding p16), two key mediators of senescence‐associated cell cycle arrest (Rayess et al. 2011; Nagano et al. 2016), was significantly downregulated following rapamycin treatment (ctrl: 0.040 ± 0.003 RPM vs. rapa: 0.031 ± 0.003 RPM, *p* < 0.05 and ctrl: 2.23 ± 0.12 × 10^−4^ RPM vs. rapa: 1.70 ± 0.18 × 10^−4^ RPM, *p* < 0.05, respectively; Figure 6E). No changes were observed in the expression of *Cdkn1a* (p21) or *Trp53* (p53), both central to DNA damage responses and stress‐induced senescence (Figure 6F) (Engeland 2022). Together, these results suggest that, while rapamycin does not fully reverse markers of T cell aging, it significantly down‐regulates key senescence‐associated genes, indicating a broader attenuation of immune aging.

## 4 Discussion

As most CVD patients are of advanced age, it is essential to account for age‐associated immune alterations when investigating potential therapies for atherosclerosis. Immunosenescence, characterized by immune dysfunction and chronic inflammation, exacerbates atherosclerosis and plaque instability, emphasizing the need for immunomodulatory interventions in this patient population. We therefore studied the therapeutic potential of rapamycin to modulate the immune system and plaque inflammation in aged atherosclerotic male *Ldlr* ^*−/−*^ mice.

Rapamycin limited plaque inflammation in aged atherosclerotic mice by reducing macrophage content, which aligns with previous studies showing that mTOR inhibition can decrease myeloid accumulation in atherosclerotic plaques, potentially by reducing monocyte recruitment from the circulation and inhibiting macrophage survival and differentiation (Verheye et al. 2007; Ai et al. 2014). In the aortic arch, macrophage numbers remained unchanged, but polarization shifted towards *Il1b* ^*+*^ macrophages, with an overall decrease in anti‐inflammatory macrophages. Although rapamycin inhibits glycolysis, a key metabolic pathway for pro‐inflammatory macrophages (Viola et al. 2019), previous studies have also reported an increased M1/M2 ratio due to increased apoptosis in M2‐like, but not M1‐like macrophages following rapamycin treatment in both mice and humans (Collins et al. 2021; Mercalli et al. 2013). In line with the increase in *Il1b* ^*+*^ macrophages, Mercalli et al. (2013) also observed elevated IL‐1β secretion upon rapamycin treatment. However, the inflammatory gene signature of aortic M1‐like macrophages was reduced, which might be attributed to the transcriptional downregulation of survival and activation pathways in rapamycin‐treated macrophages (Ko et al. 2017). We also revealed a significant reduction in neutrophils following rapamycin treatment at both cellular and single‐cell transcriptional levels. While the effects of rapamycin on neutrophils are less extensively studied, impaired neutrophil migration has been observed following rapamycin administration in vitro (Gomez‐Cambronero 2003). Since neutrophils can recruit various immune cells (Soehnlein et al. 2009; Shrestha and Hong 2023), including monocytes, their decreased presence might contribute to the observed reduction in plaque macrophage content.

Rapamycin showed the most pronounced impact on the T cell compartment in aged atherosclerotic mice, promoting a relative increase in atheroprotective Tregs at the cost of effector memory CD4^+^ T cells and elevated expression of genes crucial for their immunosuppressive function, including *Foxp3* and *Tgfb*. These results support prior work showing that mTORC1 inhibition dampens T cell activation and differentiation but favors Treg stability and expansion via metabolic reprogramming (Battaglia et al. 2005; Kopf et al. 2007; Pollizzi and Powell 2014). This finding is particularly relevant given the well‐established role of Tregs in attenuating atherosclerosis through suppression of pro‐inflammatory immune responses and stabilization of plaques (Foks et al. 2015). Notably, the observed increase in Tregs may help explain the marked reduction in macrophage abundance and inflammation within the aortic root of rapamycin‐treated mice, since lesional Tregs can suppress MCP‐1 expression and monocyte attraction (Subramanian et al. 2013).

Aligning with previous studies indicating that rapamycin can limit CD8^+^ T cell accumulation in sites of chronic inflammation (Wu et al. 2025), rapamycin treatment reduced CD8^+^ T cells in the aortic arch and LN. Nevertheless, we report an enrichment in central memory CD8^+^ cells, which has also been documented in murine infection models, where rapamycin was shown to promote the formation of memory precursors by attenuating mTORC1‐driven effector differentiation (Araki et al. 2009; Chen et al. 2023). Both on transcriptomic as well as cellular levels, we observed a decrease in double‐positive T cells, which we have previously identified within atherosclerotic tissues (de Mol et al. 2025). The precise origin and function of these cells remain unclear, but they have possibly escaped thymic selection or maturation (Winkels et al. 2021), indicating that mTOR inhibition might influence thymic output or the selection process (Almarzooqi et al. 2022; Al‐Hammadi et al. 2016).

Building on the observed changes in T cells, we found that rapamycin treatment impaired the Tfh‐GC B cell axis, reinforcing the notion that rapamycin impairs antigen‐driven humoral responses. These results are in line with prior work, showing that mTORC1 signaling is essential for GC proliferation and plasma cell differentiation (Li et al. 2017; Ye et al. 2017). The relative proportion of splenic GC B cells increased, which may reflect a compensatory expansion in response to impaired peripheral GC output or reduced migration dynamics from the spleen (Mejia et al. 2017; Zhang et al. 2010). Interestingly, we identified aortic B cell populations expressing genes associated with GC B cells. Although we did not directly assess the presence of artery tertiary lymphoid organ (ATLOs) in the current study, these findings suggest that local antigen‐driven B cell responses may occur within the diseased vessel wall. Future studies will be required to determine whether ATLO‐like structures are present in aged *Ldlr* ^*−/−*^ mice and contribute to the observed B cell phenotypes. Additionally, Wu et al. (2019) have recently shown that ABCs exhibit heightened mTORC1 activity, which supports their metabolic demands and inflammatory functions. The observed reduction in ABCs after rapamycin treatment therefore suggests a direct effect of mTORC1 inhibition on this pro‐inflammatory B cell subset. These shifts point to a dampening of both adaptive and age‐associated B cell responses.

In line with the suppressive effects of rapamycin on cellular senescence (Demidenko et al. 2009), we observed decreased immunosenescence scores for aortic leukocytes. The increase in CD27^−^ CD4^+^ T cells following rapamycin treatment, however, may represent a shift in T cell differentiation towards more terminally differentiated phenotypes, rather than a strict acceleration of senescence per se, underscoring the complexities in studying immunosenescence. Nevertheless, the reduction in key senescence‐associated genes *p16* and *Btg2*, but not *p21* and *p53* (Kumari and Jat 2021), indicates that rapamycin is able to downregulate part of the molecular signatures typically associated with cellular senescence.

Although total plasma cholesterol levels were elevated upon rapamycin treatment, consistent with previous atherosclerosis studies in young mice (Gadioli et al. 2009), plaque burden remained stable, suggesting that the immunomodulatory effect of rapamycin mitigated the pro‐atherogenic increase in cholesterol levels. Moreover, treatment was initiated in mice with advanced, established atherosclerosis and continued for 8 weeks. While this regimen was sufficient to induce profound immune remodeling and reduce plaque inflammation, a longer treatment duration or intervention at an earlier stage of disease may be required to elicit measurable effects on plaque burden. This raises the possibility that combining rapamycin with lipid‐lowering therapies such as statins could further optimize cardiovascular outcomes (Song et al. 2021).

Several limitations of this study should be acknowledged. Although plaque burden in the aortic root was unaffected by rapamycin treatment, lesion size was not assessed in the aortic arch, as we used this tissue for detailed immune profiling using scRNAseq. Therefore, we cannot exclude the possibility that local differences in plaque size contributed to the observed changes in immune cell composition, or conversely, that the reduced immune cell infiltration following rapamycin treatment was accompanied by a reduction in lesion size within the aortic arch. Although we observed marked reductions in germinal center B cells by flow cytometry and single‐cell RNA sequencing, germinal center structures were not assessed histologically in lymphoid organs. Therefore, our conclusions regarding germinal center responses are based on cellular and transcriptional analyses rather than direct tissue‐level evaluation.

Collectively, our data suggests that rapamycin exerts multifaceted immunomodulatory effects in age‐induced atherosclerosis, with pronounced inhibitory effects on adaptive immunity. Rapamycin reduced plaque inflammation, diminished pro‐inflammatory T cell responses, lowered age‐associated B cell frequencies, and attenuated senescence‐associated immune signatures, supporting its potential as an immunomodulatory strategy in aged atherosclerosis. However, translation to elderly patients requires careful consideration, as rapamycin also broadly suppressed adaptive immune responses, including germinal center B cells, plasma cells, and antigen‐driven humoral immunity, which could potentially impair protective immune responses. Furthermore, rapamycin increased circulating cholesterol levels, suggesting that combination with lipid‐lowering therapies such as statins may be required in cardiovascular disease patients. Future studies should therefore determine whether the beneficial immunomodulatory effects of rapamycin can be harnessed while minimizing unwanted effects on host defense and metabolic homeostasis. In addition, future studies should investigate how aging affects the expression and, more importantly, activity of mTOR pathway components across distinct immune cell populations on protein level, to further define the mechanisms underlying age‐dependent responses to mTOR inhibition. Taken together, our findings highlight the therapeutic potential of immune rejuvenation strategies in aged cardiovascular patients and underscore the value of targeting systemic immune compartments to influence local vascular inflammation.

## Author Contributions

J.M. and A.C.F. participated in the conceptualization, designed the figures and drafted the manuscript. J.M., F.H.S. and D.H.K. performed data analysis. J.M., F.H.S., V.S., M.A.C.D., M.N.A.B.K., P.J.S., C.J.B., and A.C.F. executed the animal experiments. F.P. and C.J.B. generated data and performed data analysis. All authors provided feedback on the research, analyses and manuscript.

## Funding

This work was supported by the Dutch Heart Foundation grant number 2018T051 to A.C.F., the ERA‐CVD B‐eatATHERO consortium; Dutch Heart Foundation grant number 2019T107 to A.C.F. 2023T138, the project “B‐specific” funded by the European Union under Grant Agreement No. 101115159; the AtheroNETH consortium, Dutch Heart Foundation grant number 01‐001‐2024‐0601; the Treat‐ATHERO consortium, Dutch Heart Foundation grant number 02‐001‐2023‐0138 to A.C.F, and MARGINALIZE‐MI with file number era4healthcvd‐105 of the research program Era4Health CARDINOVV which is (partly) financed by the Dutch Research Council (NWO).

## Conflicts of Interest

The authors declare no conflicts of interest.

## Supporting information


**Figure S1:** Gating strategy of aortic CD45^+^ cells and quality control for single‐cell RNA‐sequencing of aortic CD45^+^ cells. (A) Gating strategy of alive aortic CD45^+^ cells from control‐ and rapamycin‐treated *Ldlr* ^*−/−*^ mice. (B) Sequencing parameters from single cell CD45^+^ suspensions loaded on the 10× Genomics Chromium scRNA‐seq platform. (C) Plots showing percentage of mitochondrial genes (% mito.genes) in relation to number of genes. (D) Number of genes, and percentage of mitochondrial genes and ribosomal proteins (expressed as % of all genes) in the single cells.

**Figure S2:** Relative distribution of aortic leukocytes in rapamycin‐treated *Ldlr* ^*−/−*^ mice. Using flow cytometry, we quantified the relative distribution of CD11b^+^ myeloid cells, F4/80^+^ macrophages, Ly6G^+^ neutrophils, CD19^+^ B cells and CD4^+^, CD8^+^, and CD4^+^ CD8^+^ T cells as percentage of CD45^+^ leukocytes. Data are from *n* = 12 mice per group. Mean ± SEM plotted. Statistical significance was tested using a *t*‐test. *p < 0.05.

**Figure S3:** Single‐cell RNA‐sequencing on aortic leukocytes of *Ldlr* ^*−/−*^ mice. UMAP plots split by (A) immune cell clusters in control‐ and rapamycin‐treated *Ldlr* ^*−/−*^ mice. (B) Average expression of immune cell lineage markers mapped onto the UMAP plot. (C) Dot plot showing the average expression of myeloid cluster‐defining markers for each myeloid cluster. (D) Average expression of *Fcer2a* and *Cr2* mapped onto the UMAP plot of the B cell reclustering and (E) dot plot showing the average expression of B cell cluster‐defining markers for each B cell cluster. (F) Average expression of *Cd4* and *Cd8a* mapped onto the UMAP plot of the T cell subclustering and (G) dot plot showing the average expression of T cell cluster‐defining markers for each T cell cluster.

**Figure S4:** Rapamycin alters atherosclerosis‐specific immunoglobulin levels. The level of antibodies against the oxidation‐specific epitopes malondialdehyde (MAA‐BSA) and oxidized‐LDL (CuOxLDL) was measured in the serum. Data are shown as relative light units (RLU)/100 ms. Representative dot plots are shown. Data are from *n* = 12 mice per group. Mean ± SEM plotted. Statistical significance was tested using a *t*‐test. *p < 0.05, **p < 0.01, ***p < 0.001.

**Figure S5:** Rapamycin does not affect serum cytokine levels. Serum cytokine levels were measured using a Mouse Th‐cytokine LEGENDplex panel (12‐plex). Mean ± SEM plotted. Statistical significance was tested using a *t*‐test.

**Table S1:** Antibodies for flow cytometry.
**Table S2:** Primers for qPCR.
**Table S3:** Top 25 differentially expressed genes main clustering.
**Table S4:** Top 25 differentially expressed genes myeloid subclustering.
**Table S5:** Inflammatory genes.
**Table S6:** Top 25 differentially expressed genes B cell subclustering.
**Table S7:** Top 25 differentially expressed genes T cell subclustering.

## Data Availability

Data is available upon reasonable request to a.c.foks@lacdr.leidenuniv.nl.
