# Supplementary material for: Therapeutic Immune Reprogramming by Rapamycin Attenuates Plaque Inflammation and Lymphoid Immune Responses in Aged Atherosclerotic Mice
Source: Aging Cell. 2026 Sep 23;25(10):e70730. doi: 10.1111/acel.70730 (PMC13599350; doi:10.1111/acel.70730)
Supplement: Supplementary file 1 [file ACEL-25-e70730-s001.pdf]

Supplementary material

Therapeutic immune reprogramming by rapamycin limits plaque inflammation in aged atherosclerotic mice

Running title: *Rapamycin limits plaque inflammation*

Jill de Mol, Msc.[1], Frank H. Schaftenaar, PhD[1], Daphne H. de Korte, BSc.[1], Virginia Smit, PhD[1], Marie AC. Depuydt, PhD[1], Mireia NA. Bernabé Kleijn, BSc.[1], Peter J. van Santbrink[1], Amanda C. Foks, PhD[1]

[1] Leiden Academic Centre for Drug Research, Division of BioTherapeutics, Leiden University, Einsteinweg 55, 2333 CC Leiden, The Netherlands.

Correspondence to:

Amanda C. Foks, PhD

Division of BioTherapeutics

Leiden University, LACDR

Einsteinweg 55, 2333CC Leiden

The Netherlands

Tel; +31 (0) 71-5276213

a.c.foks@lacdr.leidenuniv.nl

Supplementary Table S1. Antibodies for flow cytometry

Marker	Fluorochrome	Clone	Cat. No.	Working concentration	Source
CD45	AF700	30-F11	103128	1:2000	Biolegend
CD44	APC	IM7	17-0441-82	1:1000	eBioscience
IL-10	APC	JES5-16E3	505010	1:100	Biolegend
CD43	APC	S11	143208	1:500	Biolegend
CXCR5	BV421	L138D7	145512	1:100	Biolegend
CD21	BV421	7E9	123422	1:400	Biolegend
TIGIT	BV421	1G9	142111	1:400	Biolegend
CD4	BV510	RM4-5	100559	1:500	Biolegend
B220	BV510	RA3-6B2	103248	1:400	Biolegend
CD19	BV605	6D5	115540	1:500	Biolegend
CD11b	BV605	M1/70	101257	1:500	Biolegend
CD62L	BV605	MEL-14	104438	1:500	Biolegend
F4/80	BV650	BM8	123149	1:500	Biolegend
RORγT	BV650	Q31-378	564722	1:300	BD Horizon
IFNγ	BV650	XMG1.2	505832	1:500	Biolegend
CD27	BV650	LG-3A10	124233	1:500	Biolegend
FoxP3	eFluor 450	FJK-16s	48-5773-82	1:500	eBioscience
Ki-67	FITC	SolA15	11-5698-82	1:1000	eBioscience
CD1d	FITC	1B1	123507	1:400	Biolegend
CD11c	FITC	N418	117306	1:500	Biolegend
CD23	PE	B3B4	101607	1:500	Biolegend
CD19	PE-Cy7	6D5	115519	1:500	Biolegend
T-bet	PE-Cy7	eBio4B10	25-5825-82	1:1000	eBioscience
GL7	PE-Cy7	GL-7	144620	1:500	Biolegend
Ly6G	PerCP	1A8	127654	1:500	Biolegend
CD5	PerCP	53-7.3	100616	1:400	Biolegend
PD-1	PerCP-eF710	J43	46-9985-80	1:400	eBioscience
CD8a	PE-Texas red	53-6.7	MCD0817	1:2000	Invitrogen
CD16/32 (Fc Block)	-	93	553142	1:250	BD Biosciences
Fixable viability dye	eFluor-780	-	65-0865-18	1:2000	eBioscience

Supplementary Table S2. Primers for qPCR

Gene	Forward primer	Reverse primer
BTG2	aaggttttcagtagggcgctccag	tggccaccttgctgatgatggg
Il-21	gccaaactcaagccatcaaaccctg	tttctcatacgaatcacaggaagggca
p16	tctttgtgtaccgctgggaacgtc	agctaagaagaaaaaggcgggctgag
p21	ctgtcttgcactctggtgtctgagc	aagaccaatctgcgcttggagtgat
p53	taaacgcttcgagatgttccgggag	aggccccactttcttgaccattgtt
36B4	taaccctgaagtgctcgacatcacag	gacgcgcttgtacccattgatgatg
Ppia	gaaaactttcgagctctgagcactgg	ctgccgccagtgccattatgg
Actin-beta	cttctttgcagctccttcgttgccg	aatacagcccggggagcatcgtc

Supplementary Table S3. Top 25 Differentially Expressed Genes Main Clustering

Cluster 0	Cluster 1	Cluster 2	Cluster 3	Cluster 4	Cluster 5	Cluster 6
Ebf1	Cd79a	Ebf1	Ebf1	Nkg7	Gm2682	Arpp21
Bank1	Ebf1	Bank1	Bank1	Ccl5	Igfbp4	2610307P16Rik
Cd79a	Bank1	Cd79a	Cd79a	Ms4a4b	Lef1	Gm4258

Cd83	Ms4a1	Mef2c	H2-Eb1	Gzmk	Ms4a4b	Dntt
Mef2c	Pax5	H2-Eb1	Mef2c	Gm2682	Prkcq	Rag1
Myo1e	Mef2c	Ms4a1	H2-Aa	Tox	Il7r	Ccr9
H2-Eb1	Cd79b	Cd79b	H2-Ab1	Ctla2a	Dapl1	Camk4
H2-Ab1	Iglc2	H2-Aa	Cd74	AW112010	Itk	Ssbp2
H2-Aa	Blnk	H2-Ab1	Pax5	Prkch	Scml4	Glcci1
Pax5	H2-DMb2	Pax5	Cd83	Ptpn22	Sidt1	Cd8b1
Ms4a1	Myo1e	Iglc2	Ighd	Atxn1	Prkch	Plxdc2
H2-DMb2	H2-Ab1	Ighd	H2-DMb2	Runx3	Txk	Tcf7
Iglc2	Iglc3	H2-DMb2	Bach2	Skap1	Emb	Aqp11
Plaur	H2-Eb1	Ly6d	Ralgps2	Cd3g	Ms4a6b	Znrf1
Ighd	Ighd	Ighm	Ighm	S100a6	Gramd3	Myb
Ly6d	Ly6d	Ralgps2	Gm31243	Cxcr6	Inpp4b	Cd4
Cd79b	Bcl11a	Iglc3	Igkc	Hip1	Grap2	Mad1l1
Gm31243	H2-Aa	Cd74	Fchsd2	Cd3e	S1pr1	Themis
Cd86	Wdfy4	Fcmr	Rel	Trac	Utrn	Cd247
Blnk	Gm31243	Blnk	Myo1e	Hcst	Dgka	Endou
Ighm	Fcmr	Cd55	Foxp1	H2-Q7	Sntb1	Bcl11b
Ralgps2	Ralgps2	Bcl11a	Fcmr	Itga4	Dusp10	Sox4
Cd74	Cd83	Gm31243	Cd79b	Rgs1	Npc2	Mier1
Lyn	Bcar3	Fchsd2	Lyn	Fyn	Peli1	Rmnd5a
Rel	Ighm	Bach2	Aff3	Ms4a6b	Rab3ip	Colq

**Cluster 7**	**Cluster 8**	**Cluster 9**	**Cluster 10**	**Cluster 11**	**Cluster 12**	**Cluster 13**
Arpp21	Ly6c2	Nkg7	Ctla4	Adgre4	C1qa	Mki67
Gm4258	Sidt1	Ccl5	Sntb1	Fcer1g	C1qb	Nusap1
2610307P16Rik	Gm2682	Gzmb	Icos	Slc8a1	C1qc	Kif11
Themis	Ccl5	Itga1	Inpp4b	Lst1	Mafb	Top2a

Camk4	Nkg7	Gm2682	Gm20275	Gngt2	Tanc2	Birc5
Ssbp2	Il7r	Ms4a4b	Rora	Lrmda	Cd14	Ube2c
Ccr9	Ms4a4b	Cd226	Izumo1r	Lyz2	Adgre1	Cdca8
Cd247	Il2rb	Klrd1	Maf	Tyrobp	Pid1	Cenpf
Znrf1	Fgf13	S100a6	Tnfsf8	Pid1	Fcer1g	Prc1
Mier1	Pde2a	AW112010	Tbc1d4	Cybb	Lgmn	Tpx2
Rag1	Txk	Cxcr6	Gpm6b	Ear2	Dab2	Lockd
Glcci1	Lef1	Id2	Ptpn13	Ms4a6c	Gab2	Pclaf
Tcf7	Hcst	Ctsw	Cd40lg	Fgr	Ms4a7	Diaph3
Mad1l1	Ms4a6b	Klrk1	Tnfrsf4	Cd300a	Cd63	Knl1
Dntt	Gramd3	Cd3g	Gm49180	Pla2g7	Frmd4b	Ccna2
Bcl11b	1700025G04Rik	Prkch	Nrn1	Clec4a3	Tyrobp	Cdca3
Cd4	Prkcq	Hcst	Itgb8	Gsr	C5ar1	Ccnb2
Plxdc2	Klrd1	Atxn1	Sostdc1	Ctsb	Zfhx3	Incenp
Skap1	AW112010	Itgb1	Dgki	Zeb2	Creb5	Smc2
Cd8b1	Ctla2a	H2-Q7	8030451A03Rik	Dusp16	Cxcl16	Ect2
Satb1	Emb	Hip1	Foxp3	Cebpb	Hexb	Kif15
Rmnd5a	Ust	Gzmk	Il21	Cd300c2	Csf1r	Cks2
Slc9a9	Atp8b4	Chn2	Prkca	Sirpa	Ctsb	Racgap1
Trbc2	Il18r1	Gm44174	Ikzf2	Gab2	Basp1	Cdk1
Myb	Ptpn22	Gzma	Lrig1	Spi1	Apoe	Cenpe

**Cluster 14**	**Cluster 15**	**Cluster 16**	**Cluster 17**	**Cluster 18**	**Cluster 19**	**Cluster 20**
Il1b	Il1b	Ly6c2	Ifitm3	Pid1	Large1	Pclaf
Cxcl2	Csf3r	Sidt1	Plbd1	Plcb1	Il23r	Mcm6
Tyrobp	S100a8	Gm2682	Ifitm2	Ifitm3	St6galnac3	Mcm7
Il1r2	S100a9	Nkg7	S100a4	Lyz2	Rora	Rrm2
Fcer1g	Il1r2	Il2rb	Cd300a	Ms4a6c	Igf1r	Hells

C5ar1	Hdc	Klre1	H2-DMb1	Fn1	Tmem176b	Dut
Ier3	Slpi	Ccl5	Alox5ap	Tgfbi	Tmem176a	Dtl
Clec4d	Tyrobp	Klrk1	Fcer1g	Ccr2	Il1r1	Mcm3
Clec4e	Clec4d	Ms4a4b	Pid1	Lgals3	Cxcr6	Stmn1
Csf3r	Gsr	Klrd1	Tyrobp	Gda	Zbtb16	Uhrf1
Cd9	Cxcl2	1700025G04Rik	Slc8a1	Zeb2	Il7r	Pola1
Ccrl2	Slc7a11	Ctla2a	Syngr2	Ifitm2	Il18r1	Dntt
Acod1	Ifitm2	Il18r1	Gm2a	Fcer1g	Pard3	Cdca7
Slc7a11	Retnlg	Klrc1	Wfdc17	Tyrobp	Clnk	Gmnn
Cebpb	Acod1	Pde2a	Spi1	Mpeg1	Maf	Ezh2
Hdc	Msrb1	Klrc2	Ms4a6c	App	Cd163l1	Lig1
Gsr	Fcer1g	Klra7	Ifi30	Alox5ap	Trdc	Hmgn2
Egr1	Ccl6	Samd3	Lgals3	S100a4	Tcrg-C1	Tipin
S100a8	C5ar1	Xcl1	Alcam	Wfdc17	Adam12	Siva1
Nlrp3	Alox5ap	Yes1	Skap2	Slc8a1	Zfp462	Tyms
Alox5ap	Mxd1	Cd7	Trerf1	Ccl6	Il2ra	Pcna
S100a9	Lilr4b	Ifngas1	Fgr	Lrmda	Ckb	Top2a
Cd14	Lmnb1	Klrb1c	Ctsh	Fgr	Plcb4	Tfdp1
Dusp1	Gda	Clnk	Dock4	Emilin2	Cdh10	Nsd2
Plek	Cebpb	Itga2	Cbfa2t3	Cybb	Kcnk1	Zfp367

**Cluster 21**	**Cluster 22**	**Cluster 23**	**Cluster 24**	**Cluster 25**	**Cluster 26**
Sox5	Wfdc21	Chl1	2610307P16Rik	Igfbp4	Gzmk
Mzb1	Lcn2	Itm2a	Arpp21	Dapl1	Nkg7
Cacna1e	Retnlg	B630019A10Rik	Gm4258	Gm2682	Ccl5
C130026I21Rik	Mmp9	2610307P16Rik	Rag1	Inpp4b	Cxcr6
Iglc1	Anxa1	Tox	Ccr9	Pax5	Itga1
Pard3b	Hp	Lef1	Dntt	Lef1	Hip1

Fcrl5	Ngp	Ccr9	Colq	Rapgef4	Epha3
Bhlhe41	Mgst1	Ccr4	Glcci1	Ccr7	Gzmb
Il5ra	Pglyrp1	Camk4	Satb1	Bach2	Pdcd1
AC125149.3	Mmp8	Satb1	Myb	Auts2	Ms4a4b
Dnase1l3	Ifitm6	Cd28	Mad1l1	Bank1	Atxn1
1810046K07Rik	S100a8	Gm4258	Themis	Scml4	Gm44174
Gm30382	S100a9	Tcf7	Camk4	Cpm	Bank1
Nid1	Slpi	Sox4	Cd4	Ebf1	Ctsw
Zbtb32	Alox5ap	Trbc2	Sox4	Cd79a	Cish
Gm47957	Pbx1	Cd5	Endou	Usp28	Ctla2a
AC133103.1	Gsr	Cd247	Ssbp2	Pced1b	AW112010
AC168977.1	Gda	Zcchc12	Chrna9	Sntb1	Gm2682
Sox5os4	Msrb1	Skap1	Mier1	Cd55	Ifng
Robo1	Ly6g	Znrf1	Plxdc2	St6gal1	Xcl1
Diras2	Cd177	Slc9a9	Wdr78	S1pr1	Tmem163
Gnb3	Lyz2	Trac	Tcf12	Bcar3	Ebf1
Gm26704	Tyrobp	Bcl11b	Aqp11	Slamf6	Abcb1a
Lmo7	Itgam	Tnik	Bank1	1700062C10Rik	Tbx21
Oosp1	Hdc	4930417O13Rik	Kremen1	Sh3bp5	Sntb1

**Cluster 27**	**Cluster 28**	**Cluster 29**	**Cluster 30**	**Cluster 31**	**Cluster 32**
Ncr1	Il2ra	Basp1	2610307P16Rik	Mitf	Ly6c2
Klrb1c	Hes1	Jchain	Ccr9	Mpeg1	Sidt1
Klre1	Tcrg-C1	Eaf2	Arpp21	Sirpa	1700025G04Rik
Klrk1	Gm4632	Rgs13	Gm4258	Cd68	Yes1
Il2rb	Myl10	Mybl1	Rag1	Anxa4	Il18r1
Clnk	Tcrg-C2	Mef2b	Nkg7	Lgals3	Klra7
Gzma	Notch1	Cecr2	Ccl5	Lrp1	Pde2a

Klrc2	Tsc22d1	Slc41a2	Camk4	Plin2	Ifngas1
Serpinb9	5830418P13Rik	Hmgn3	Cd4	Cd300a	Il2rb
Prf1	Tcrg-C4	Nuggc	Themis	Pla2g7	Samd3
Itga2	Gria3	Scimp	Ctla2a	Clec4n	Ccl5
Klrb1b	Zfp704	Aicda	Myb	Slc8a1	Il7r
Aoah	Notch3	Neil1	Ssbp2	Cd300c2	Btbd11
Styk1	Angpt1	Smagp	Dntt	Cd63	Fgf13
Serpinb6b	Gfra1	Pclaf	Ms4a4b	Ninj1	Gm2682
Klri2	Ccdc18	Mki67	Nav2	Myof	Klrc2
Klra8	Ndrg1	H1fx	Sla2	Mmp14	Gzmm
Spry2	Nckap5	Smco4	Ntrk3	Tgfbi	Ctsw
Klrb1f	Trgv2	Sox5	Cd247	Grn	Klrc1
Klra4	Epb41l4b	Asf1b	Colq	Tnfaip2	Ctla2a
Adamts14	Adamts17	Mbd4	Tcf7	Mafb	Klre1
Klra7	AW011738	Rassf6	Cd8a	Csf1r	Ust
Klra9	Gm2629	Hpse	Mad1l1	Clec4d	Klrd1
Klrb1a	Tmem121	Ighg1	Cxcr6	Fnip2	Nkg7
Cma1	Ptcra	S1pr2	Chrna9	F10	Eomes

Cluster 33	Cluster 34	Cluster 35	Cluster 36	Cluster 37	Cluster 38	Cluster 39
Fn1	Camp	Mreg	Wfdc21	Ifi205	C1qa	Csf3r
Mrc1	Ngp	Fscn1	Lcn2	Naaa	C1qb	Adgre4
Retnla	Ighg2b	Anxa3	Ngp	Plbd1	C1qc	Tgm2
Fcrls	Jchain	Tbc1d8	Cd177	Naga	Cd5l	Hdc
Gfra2	Igkc	Cacnb3	Ifitm6	Shtn1	Ccl8	Ear2
Aldh1a2	Ighg2c	Tbc1d4	Ltf	Ckb	Ms4a7	Trem1
Kcnq1	Lyz2	Nav1	Camp	Fmnl2	Apoe	Zfyve9
9530059O14Rik	Lcn2	Tmtc2	Chil3	Clec9a	Cd63	Treml4

Hr	Fth1	Tnfrsf11a	Dach1	Olfm1	Mmp12	Ptgs2
Tmem229a	Igha	Cxcl16	Mgst1	Tbc1d9	Lyz2	Ace
Plcb1	Gm42418	Strip2	Anxa1	Rab7b	Trem2	Cd300e
Pid1	Tmsb4x	Tmem176a	Hp	Ccser1	Wfdc17	Acod1
Lpar1	Fcer1g	Socs2	Adpgk	Htr7	Igf1	Nlrp3
Gm45238	Ptma	Rogdi	Mcemp1	Tbc1d8	Gpnmb	Pparg
Dock1	Spp1	Slc22a23	Clec4a2	Gatm	Ctsb	Apoc2
Rem1	Ltf	Zbtb46	Trem3	Flt3	Il18bp	Clec4d
Apba1	Ighg1	Zfp366	Lrg1	BC028528	Selenop	Hcar2
Lyz1	H2afz	Frmd4a	Pygl	St3gal5	Gas6	Gm5150
Stxbp6	C1qa	Zmynd15	Ly6g	Gm36723	Pf4	Il1r2
Thsd4	Stmn1	Flt3	Ckap4	Batf3	Aif1	Cd36
Tbxas1	Apoe	Fnbp1l	Hdc	Pak1	Ftl1	Clec4e
9530036M11Rik	H2-K1	Slco5a1	Sgms2	Sept3	Fcer1g	Il1rn
E230016K23Rik	C1qc	Nostrin	G0s2	Xcr1	Lgals3	Skint3
Fgfr1	H2-Eb1	Tmem150c	Igsf6	Aif1	Syngr1	Sirpb1c
Lgi3	C1qb	Adgrg6	Anxa3	Srgap3	Fth1	Ifitm1

**Cluster 40**	**Cluster 41**	**Cluster 42**	**Cluster 43**	**Cluster 44**	**Cluster 45**
Ccnb2	Mcm5	Jchain	Siglech	Cplx2	Slc24a3
Cenpf	Hells	Derl3	Grm8	Igll1	Cd200r3
Stmn1	Rad51b	Iglv1	Cox6a2	Vpreb2	Gata2
Dntt	Mcm3	Sdc1	Cd300c	Smtnl2	Cpa3
Endou	Lig1	Cpeb3	Upb1	Gm34095	Cyp11a1
Cdc20	Mcm2	Iglv3	Smim5	Gm32569	Mcpt8
Gm4258	Uhrf1	Fkbp11	Ccnd1	Ren1	Ms4a2
Cenpe	Pola1	Tnfrsf17	P2ry14	Gfra1	Fcer1a
Arpp21	Mcm6	Pon3	Gm21762	Fam189a1	Csrp3

Ptms	Clspn	Oosp1	Pdzd4	Cecr2	Il6
Hist1h2bc	Mcm7	Pls1	Lifr	Vpreb1	Il4
Mns1	Mcm4	Rhobtb1	Klra17	Tmem132e	Prss34
Rag1	Dhfr	Cacna1s	Ppfia4	P2rx3	Rnase12
Birc5	Diaph3	Prg2	Paqr5	Fmn2	1110028F11Rik
Ppp1r14b	Tipin	Cped1	Eldr	Gpat3	Gpc4
Tcf7	Tcf19	Chst1	Klk1	2310008N11Rik	Btnl9
Sox4	Rad51	Gpm6a	Pir	Tifa	Fam110c
Gm26737	Pole	Prlr	Cd209d	Vpreb3	Slc6a4
Lockd	Chaf1a	Cacna1h	Sh3bp4	Nrxn1	6030469F06Rik
Hmgb1	Brca1	Reln	Lrp8os2	Rsph1	Ptger3
Hmgn2	E2f1	Ccr10	Clec9a	Col25a1	A230098N10Rik
Cd8b1	Gins2	Pycr1	Lefty1	Akap12	Alox15
Aqp11	Gmnn	Alpl	Sh3bgr	Tmprss3	Itga2b
2610307P16Rik	Ung	Eef1akmt3	Klk1b27	Bcl7a	Hgf
Anxa2	Mcm10	Tspan15	Gm34680	Nuak1	Far2

Supplementary Table S4. Top 25 Differentially Expressed Genes Myeloid Subclustering

**Cluster 0 Nlrp3^{hi} MΦ**	**Cluster 1 DC I**	**Cluster 2 Mox MΦ**	**Cluster 3 Il1b^{hi} MΦ**	**Cluster 4 Ccr2^{hi} M**	**Cluster 5 Trem2^{hi} MΦ**	**Cluster 6 Neutro I**
Csf3r	Cd209a	Cd300e	Cxcl2	Fn1	C1qa	Mmp8
Hdc	Olfm1	Treml4	Il1b	Plcb1	C1qc	Wfdc21
Il1b	Clec10a	Ear2	Nfkbia	Vcan	C1qb	Retnlg
Il1r2	Ddr1	Adgre4	Ier3	Lyz2	Ms4a7	Lcn2
Slpi	Zbtb46	Bcl2a1a	Ccrl2	Arhgef37	Cadm1	Mmp9
Srgn	Flt3	Gngt2	Csf1	Ifitm3	C3ar1	Ngp
Retnlg	Syngr2	Bcl2a1d	Il1a	Ms4a6c	Igf1	Ly6g
S100a9	Rtn1	Pla2g7	Il1rn	Ifi204	Trem2	Cd177

Cxcr2	Gm2a	Ms4a6c	Clec4n	Ccl9	Itga9	Scrg1
S100a8	Ccnd1	Bcl2a1b	Ccl3	Ms4a8a	Nes	Ceacam10
Acod1	Bex6	Sod2	Ptgs2	Chil3	Col14a1	S100a9
Mxd1	Htr7	Pparg	Il1r2	Ifi211	Gm15155	S100a8
Clec4d	Actb	Ms4a6d	Hcar2	Tgfbi	Arhgap22	Camp
Arg2	Cfp	Clec4a1	Nlrp3	Dmkn	Zfp618	Mrgpra2b
Dusp1	Plbd1	Clec4a3	Egr1	AI839979	Mafb	Pglyrp1
Ankrd33b	Dpysl2	Fabp4	Gm31718	Lgals3	Pmp22	Ltf
Zfp36	Fh1	Dusp16	Plek	Stxbp6	Mmp12	Padi4
Cxcl2	Klri1	Prdx1	Srgn	Klra2	St18	Bmx
Gsr	Spint1	Smpdl3b	Cd14	Psap	Gatm	Adpgk
Hcar2	Lsp1	Lst1	Cxcl3	Sirpb1c	Slamf9	Mgst1
Stfa2l1	Ifitm3	Pot1b	Acod1	Ifi207	Cxcl16	Dach1
G0s2	Vrk1	Fth1	Siglecf	Emilin2	Ms4a14	Itgb2l
Msrb1	Eps8	Spred1	Clec5a	Msr1	Zmynd15	Chil1
Ptgs2	Actg1	Txnrd1	Clec4e	Mgst1	Snx24	Ifitm6
Il1f9	Dab2	Zc3h12c	Mcl1	Fcgr1	Rnf180	1700047M11Rik

**Cluster 7 Nr4a1^{hi} MΦ**	**Cluster 8 Foamy MΦ**	**Cluster 9 Res. MΦ**	**Cluster 10 Neutro II**	**Cluster 11 Mix**	**Cluster 12 cDC I**
Adgre4	Gpnmb	Mrc1	Ltf	Gm42418	Fscn1
Ace	Mmp14	Pf4	Camp	Zeb2	Cacnb3
Zfyve9	Fam20c	Gas6	Ngp	Pid1	Slc22a23
Ear2	Anxa4	C1qc	Cd177	Trps1	Slco5a1
Apoc2	Rai14	Ccl12	Chil3	Plcb1	Tmem150c
Slc12a2	Nr1h3	C1qa	Abca13	Vcan	Syn3
Gngt2	Vat1	Mir99ahg	Itgb2l	Zfp638	Arhgap28
Slc8a1	Cstb	Mgl2	Cebpe	Vps13b	Slc4a8

Pparg	Myof	Slco2b1	Ncam1	Zfand3	Gm16685
Dusp16	Mitf	C1qb	Fcnb	Tet3	Dscaml1
Treml4	Gdf15	Ophn1	Orm1	Picalm	Nudt17
Cd36	Mmp12	Cbr2	Ica1	Ext1	Gm10432
Eno3	Lpl	Plxna4	Ly6g	1700013M08Rik	Ccl22
Pot1b	Prdx1	Stab1	Lcn2	Rnf225	Gm42601
Grk3	Atp6v0c	Abca9	Cpa6	Gm10688	Gm13546
Smpdl3b	Ftl1	C3ar1	Dach1	Gm11674	Gm20465
Rap1a	Clec4n	Prune2	Wfdc21	Btbd9	Il12b
Cd300e	F10	2610203C22Rik	Prom1	Fn1	Apol7c
Grk5	Plxna1	Dab2	Adpgk	Taok3	Zmynd15
Ceacam1	Psap	Frmd4b	Olfml2b	Rrbp1	Zfp366
Ccdc192	Fth1	Cd163	4930438A08Rik	Fndc3a	Gli3
Gpr141	Ralgds	Hpgd	B230208H11Rik	Gm11672	Rasal2
Fgr	Lgals3	C4b	Ankrd22	Ccnl1	Rogdi
Ly6i	Srxn1	Pmp22	Lbp	Traj58	Tnfrsf11a
Cyp2ab1	Esd	Kitl	Ms4a3	Map4k4	Oprd1

**Cluster 13 cDCII**	**Cluster 14 Foamy MΦ**	**Cluster 15 Il1b^{hi}/Mox MΦ**	**Cluster 16 pDC**	**Cluster 17 Fn1^{hi} MΦ**	**Cluster 18 MC**
Ifi205	Cd5l	Adgre4	Siglech	Retnla	Cd200r3
Clec9a	C1qa	Ace	Grm8	Kcnq1	Cyp11a1
Ccser1	C1qc	Ear2	Cox6a2	Aldh1a2	Fcer1a
Xcr1	C1qb	Ptgs2	Cd300c	Gfra2	Mcpt8
Sept3	Apoe	Acod1	Upb1	Thsd4	Ms4a2
Tlr3	Ftl1	Cd36	Gm21762	Lyz1	Csrp3
Gm43914	Ccl8	Zfyve9	Klk1	Rarb	Il4
Snx22	Fth1	Tgm2	Klk1b27	Hr	Prss34

F930017D23Rik	Ms4a7	Treml4	Sh3bgr	9530059O14Rik	Rnase12
Gpr141b	Mmp12	Cxcl2	Cdh5	Tmem229a	1110028F11Rik
Plce1	Prdx1	Cd300e	Gm12253	Fcrls	Itga2b
P3h2	Ctsb	Nlrp3	Atp2a1	Gm45238	Gpc4
Mycl	Cd63	Pparg	Gm14964	Ms4a8a	Il6
Fam149a	Gm20478	Dusp16	Pir	Lpar1	Gm47599
Pdia5	Psap	Trem1	Cd209d	Car6	Slc6a4
Homer2	Gpnmb	Apoc2	Gm14207	Ecm1	Ctsg
Ffar4	Il18bp	Csf1	Lefty1	Cgnl1	Samd13
Dbn1	Atp6v0d2	Il1b	Fyb2	Gm41307	Fam110c
Htr7	Syt14	Hcar2	Gm829	9530036M11Rik	6030469F06Rik
Tspan33	Trem2	Csf3r	Nrxn2	Fn1	D430036J16Rik
Naaa	Slc16a9	Hdc	Klra17	Chrna5	Gm11697
Zfp366	Wfdc17	Clec4e	Fads3	Rem1	Btnl9
Rab7b	Igf1	AU019990	Rai2	Mrc1	Gna14
Tlr11	Fabp5	Ly6i	Fbxl13	Prg4	Cd200r3
Batf3	Anxa5	Gm5150	Foxr1	Ak8	Cyp11a1

Supplementary Table S5. Inflammatory Genes

Cytokines	Chemokines	TLRs	Transcription Factors	Enzymes	Receptors	Inflammasome
Tnf	Ccl2	Tlr2	Nfkb1	Nos2	Cd14	Nlrp3
Il1b	Ccl3	Tlr4	RelA	Ptgs2	Cd36	Casp1
Il6	Ccl4	Tlr7	RelB	Cybb	Cd40	Pycard
Il12a	Ccl5	Tlr9	Stat1	Sod2	Fcgr1	
Il12b	Cxcl9		Stat3	Arg1	Fcgr3	
Il18	Cxcl10		Stat6			
Il23a	Cxcl11		Irf5			
			Irf7			
			Irf8			
			Cebpb			
			Cebpd			
			Hif1a			
			Jun			
			Fos			

Supplementary Table S6. Top 25 Differentially Expressed Genes B cell subclustering

Cluster 0 B2-like I	Cluster 1 B2-like II	Cluster 2 B1/Breg	Cluster 3 Activated	Cluster 4 Memory	Cluster 5 Resting	Cluster 6 GC-like
Satb1	Cd52	Plac8	Rps2	Sox5	Hspa1a	Malat1
Junb	Fau	Pik3r4	Hsp90ab1	Rbm47	Hspa1b	mt-Atp6
Dennd4a	Rps24	Bhlhe40	Srm	Ahnak	Hsph1	Ebf1
Vps37b	Cd74	Arhgap24	Ncl	Bhlhe41	Dnajb1	mt-Co3
Btg1	Rps8	Stt3b	Ranbp1	Cd80	Dnaja1	Arhgap15
H3f3b	Cd79a	Mreg	Npm1	Pstpip2	Hsp90aa1	Bank1
Cd83	Ly6d	Atxn1	Il4i1	Gm30382	Hsp90ab1	mt-Cytb
Stk17b	Rps4x	Ccr6	Ppia	Gm47957	Hspe1	mt-Co1
Fbxo11	Tmsb10	Cd86	Ptma	Zbtb32	Hspa8	Foxp1
Lncpint	Ly6e	Gla	Mettl1	Nrp2	Ubc	Aff3
Irs2	Rps27	Dusp4	Eif5a	Tbc1d9	Ubb	Bach2
Inpp5d	Ighd	Rab30	Apex1	Itgb1	Jun	Gm31243
Nr4a1	Arhgdib	Cd69	Nme2	1810046K07Rik	Ahsa1	mt-Co2
Plaur	March1	Bcl2a1b	C1qbp	Plscr1	Ccdc117	mt-Nd1
Kras	Rpl30	Gch1	Nop58	Fcrl5	Stip1	Ptprc
Klf2	Rpl21	Eif2ak3	Eif4a1	Robo1	Lncpint	mt-Nd4
Cd86	Rps20	Rasgef1b	Nme1	Ahnak2	Hspd1	Rbm39
Cyth1	Cd79b	Cd72	Marcksl1	Atxn1	Lifr	mt-Nd2
Ccr7	Rps15a	Pkib	Mdn1	Syn3	Swt1	Ripor2
Ets1	Rpl13	St6galnac3	Ppp1r14b	Tppp3	Dedd2	Man1a
Cxcr4	Tmsb4x	Slc23a2	Set	Cd9	P4ha1	Fchsd2
Rel	Myl12b	Gem	Mif	Cd300lf	Rsrp1	Ppp3ca
Ppp1r16b	Rps21	Rps12	Ccnd2	Nid1	Mat2a	Ralgps2
Fam107b	Shisa5	Smad3	Pa2g4	Mcc	Ptges3	Foxn3
Elmsan1	Actb	Smad7	Gar1	AC125149.3	Hspa4l	Ankrd11

**Cluster 7 ABCs**	**Cluster 8 Immature**	**Cluster 9 PCs**
Basp1	Agbl1	Tent5c
Jchain	Gfra1	Creld2
Tox	Akap12	Derl3
Rgs13	Nrxn1	Iglv1
Eaf2	Map2	Creb3l2
Mybl1	Ppm1e	Sdc1
Mef2b	Serinc5	Cpeb3
Ssbp3	Prkcg	Tmem176b
Trerf1	Vpreb3	Iglv3
Ezh2	Cplx2	Fkbp11
Hmgn3	Cecr2	Sec24d
Pclaf	Zeb2	Tmem176a
Mki67	Fam189a1	Tnfrsf17
Cks2	Igll1	Cd28
Nuggc	Fam129c	Plpp5
Scimp	Gpat3	Pon3
Fas	Atp1b1	Gm5547
Anxa2	Rapgefl1	Oosp1
Aicda	Mctp2	Pls1
Smco4	Sox4	Rhobtb1
Neil1	Nuak1	Prdm1
Top2a	Myb	Ankrd55
Mcm6	Vpreb1	Prg2
Gmnn	Ighm	Cped1
Hells	Erg	Selenom

Supplementary Table S7. Top 25 Differentially Expressed Genes T cell Subclustering

**Cluster 0** **Gzmk^{+} I**	**Cluster 1** **Sell^{+}CD4^{+}**	**Cluster 2** **CD8^{+} CM I**	**Cluster 3** **Gzmb^{+} CD8^{+}**	**Cluster 4** **Sell^{+}CD4^{+}**	**Cluster 5** **CD8^{+} CM II**	**Cluster 6** **CD4^{+} EM**
Gzmk	Igfbp4	Ly6c2	Itga1	Igfbp4	Klra7	Gpm6b
Pdcd1	Dapl1	Fcgrt	Gzmb	St8sia6	Klra1	Sostdc1
Nkg7	Lef1	Yes1	Gzma	Dusp10	Klre1	Cd4
Hip1	Rps20	Rps12	Zeb2	Ly6c1	Clnk	Cacna1d
Tox	Auts2	Sidt1	Chn2	Lef1	Klrc2	8030451A03Rik
Epha3	Rplp1	Gzmm	Fgl2	Bach2	Ikzf2	Cd200
S100a6	Rps24	Rpl23	Lgals3	Satb1	Rbpms	Gm49180
Tcrg-C2	Fam241a	Bbs9	Cd226	Ifngr2	Ly6c2	Hs6st3
Cxcr6	Bach2	Rps16	Ccl5	Pdlim4	1700025G04Rik	Tnfrsf4
Cables1	Tpt1	Pde2a	S100a6	Rps29	Sidt1	Tiam1
Atxn1	Gm43698	Rpl39	Atp8a2	Myo10	Klra6	Cd40lg
S100a4	Ccr7	1700025G04Rik	Il12rb2	Tpt1	Klrb1c	Dgki
Ccl4	Rplp0	Rpl13	Nkg7	Rps8	Klrc1	Izumo1r
Tcrg-C4	Usp28	Rplp0	Klrk1	Arhgap15	Spry2	Tnfsf11
Rgs1	Rpl23	Rps19	Nebl	Foxp1	Klrk1	Eea1
Cd3g	Rps8	Ms4a4c	Itgb1	Klf2	Pde2a	Tbc1d4
Trps1	Rps29	Rplp1	Id2	Cytip	Itga2	Crppa
H2-K1	Syt6	Rplp2	Itm2b	Atp1b1	Fcer1g	Il21
AW112010	Rps4x	Rps2	Tmem163	Pecam1	Ifngas1	Maf
Adam19	Actn1	Cmah	Klrd1	Rpl35a	Ccl5	Tnc

Ccl5	Rapgef4	Satb1	AU02020 6	Rps20	Il18r1	Csgalnact1
Osbpl3	Rps27a	Rps18	Hip1	Rps26	Yes 1	Phactr2
Ctla2a	Rps7	Rps20	Styk1	Ccr7	Txk	Gm47957
Trgv 2	Rps3a1	Rps10	Tmsb4x	Rps24	Gas7	Hif1a
S100a1 1	Rpl13	Dlg5	Dennd4a	Cd4	Samd3	Ctla4

**Cluster 7** **Gzmk$^+$ II**	**Cluster 8** **Dying CD8$^+$**	**Cluster 9** **Tregs**	**Cluster 10** **CD8$^+$ CM III**	**Cluster 11** **Prolif. CD8$^+$**	**Cluster 12** **NKT**	**Cluster 13** **IFN CD8$^+$**
Gzmk	Malat1	Foxp3	Tnni1	Uhrf1	Klra9	Ifit1
Itga4	Igkc	Ikzf2	Pclo	E2f1	Zbtb16	Rsad2
Lmo1	mt-Co1	Izumo1r	Gm15353	Clspn	Klrb1c	Ifit3
Rasgef1b	mt-Co2	Plcl1	Cdhr3	Mcm10	Clnk	Cxcl10
Gm15987	mt-Co3	Dst	St3gal5	Kntc1	Tgfa	Isg15
Eomes	Skap1	Mctp1	S100a4	Rad54l	Styk1	Ifi204
Ccl5	mt-Atp6	Plcb4	Gm50034	Mcm5	Rab4a	Ifit3b
Kcnip3	mt-Cytb	Cep112	Trpc4ap	Brca1	Trf	Ifi44
Sh2d1a	Ptprc	Hdac9	Hip1r	Rad51	Kcnq5	A330040F15Rik
Fyn	Mbnl1	Gm28112	Ttc19	Cdc45	Klra7	Rxfp1
Tox	Itga4	Tnfrsf4	Rora	Chaf1a	Chn2	Acp4
Esm1	Cd74	Itgb8	Pde7b	Rad51b	Trav11	Piwil4
Ptprk	mt-Nd1	Ctla4	Zfp462	Cdc6	Epas1	Ccdc153
Ier3	mt-Nd4	Nrp1	Gm12648	Ncapg2	Fgd5	Lsmem2
Rnf43	Dock2	Cd81	Abra	Dhfr	Glis3	C330019F10Rik
Epha3	Gm2682	Il2ra	Gm16098	Mcm2	Tmem64	Ighv9-3
Ms4a4b	Arhgap15	Nav2	Dbndd1	Gins2	Cap2	Cdh2
Zfhx3	Ankrd44	Gm31718	Gm48829	Fignl1	Slc16a10	Gm47243

Tspan3	Tln1	Cd83	Cx3cr1	E2f7	Il12rb2	Prmt8
Crtam	Gnb1	Tiam1	Klf12	Brip1	Cd40lg	Ifi211
Dscam	Themis	Lrig1	Kit	Rad51ap1	Il4	Rtp4
1700029J03Rik	Hnrnpc	Ikzf4	Phlda1	Neil3	Socs2	Rnf213
Asap1	Ankrd11	Sdc4	Gm11973	Cenph	Litaf	Bst2
Il10ra	Atxn1	Slc24a3	Klrb1a	Mcm3	Dusp1	Isg20
Slc24a2	Pitpnc1	Gm3164	Phf8	Pole	Hhex	Serpina3f
