# Supplementary material for: Therapeutic Immune Reprogramming by Rapamycin Attenuates Plaque Inflammation and Lymphoid Immune Responses in Aged Atherosclerotic Mice
Source: Aging Cell. 2026 Sep 23;25(10):e70730. doi: 10.1111/acel.70730 (PMC13599350; doi:10.1111/acel.70730)
Supplement: Supplementary file 3 [file ACEL-25-e70730-s003.pdf]

A

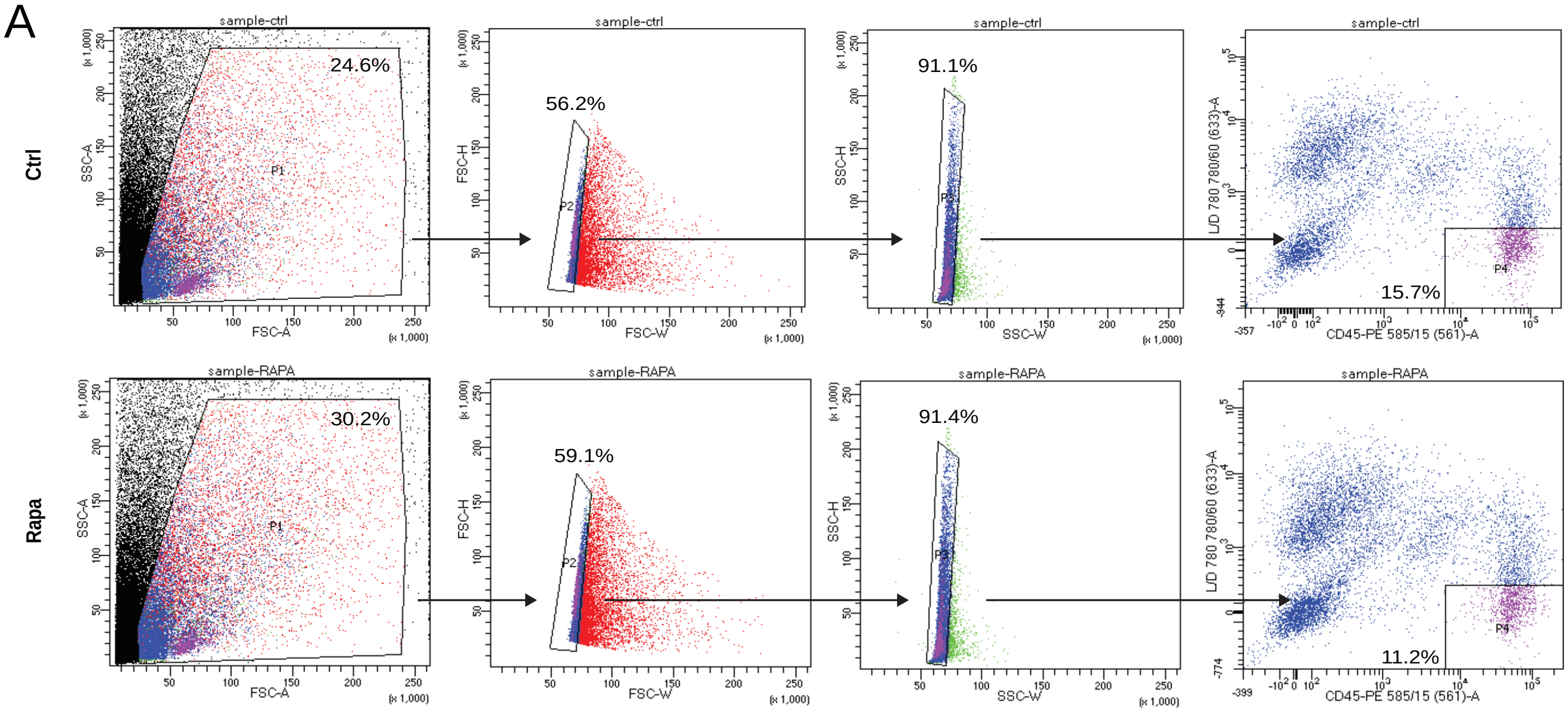

B

Metrics aortic CD45+ cells	Ctrl	Rapa
Estimated Number of Cells	19,032	19,766
Mean Reads per Cell	8,369	8,565
Median Genes per Cell	1,282	1,264
Number of Reads	159,272,751	169,292,087
Total Genes Detected	22,635	22,515
Median UMI Counts per Cell	2,334	2,435
Valid Barcodes	96.20%	96.50%
Sequencing Saturation	42.40%	45.20%
Q30 Bases in Barcode	97.20%	97.20%
Q30 Bases in RNA Read	96.20%	96.40%
Q30 Bases in UMI	97.80%	97.80%
Reads Mapped to Genome	96.50%	96.70%
Reads Mapped Confidently to Genome	94.70%	94.80%
Reads Mapped Confidently to Intergenic Regions	3.10%	3.00%
Reads Mapped Confidently to Intronic Regions	29.40%	29.60%
Reads Mapped Confidently to Extronic Regions	62.10%	62.20%
Reads Mapped Confidently to Transcriptome	81.80%	83.00%
Reads Mapped Antisense to Gene	9.40%	8.30%
Fraction Reads in Cells	89.40%	93.30%

C

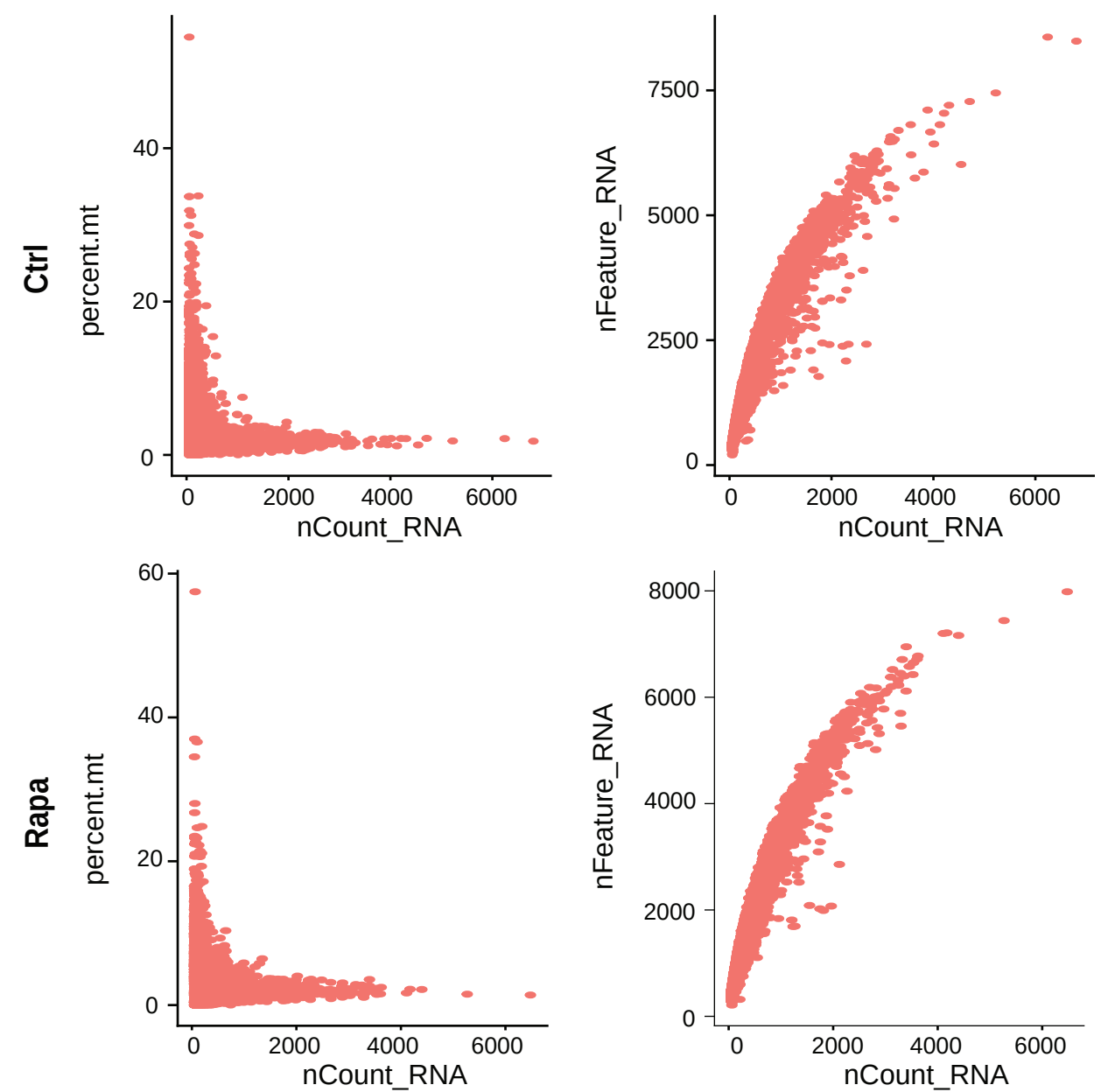

D

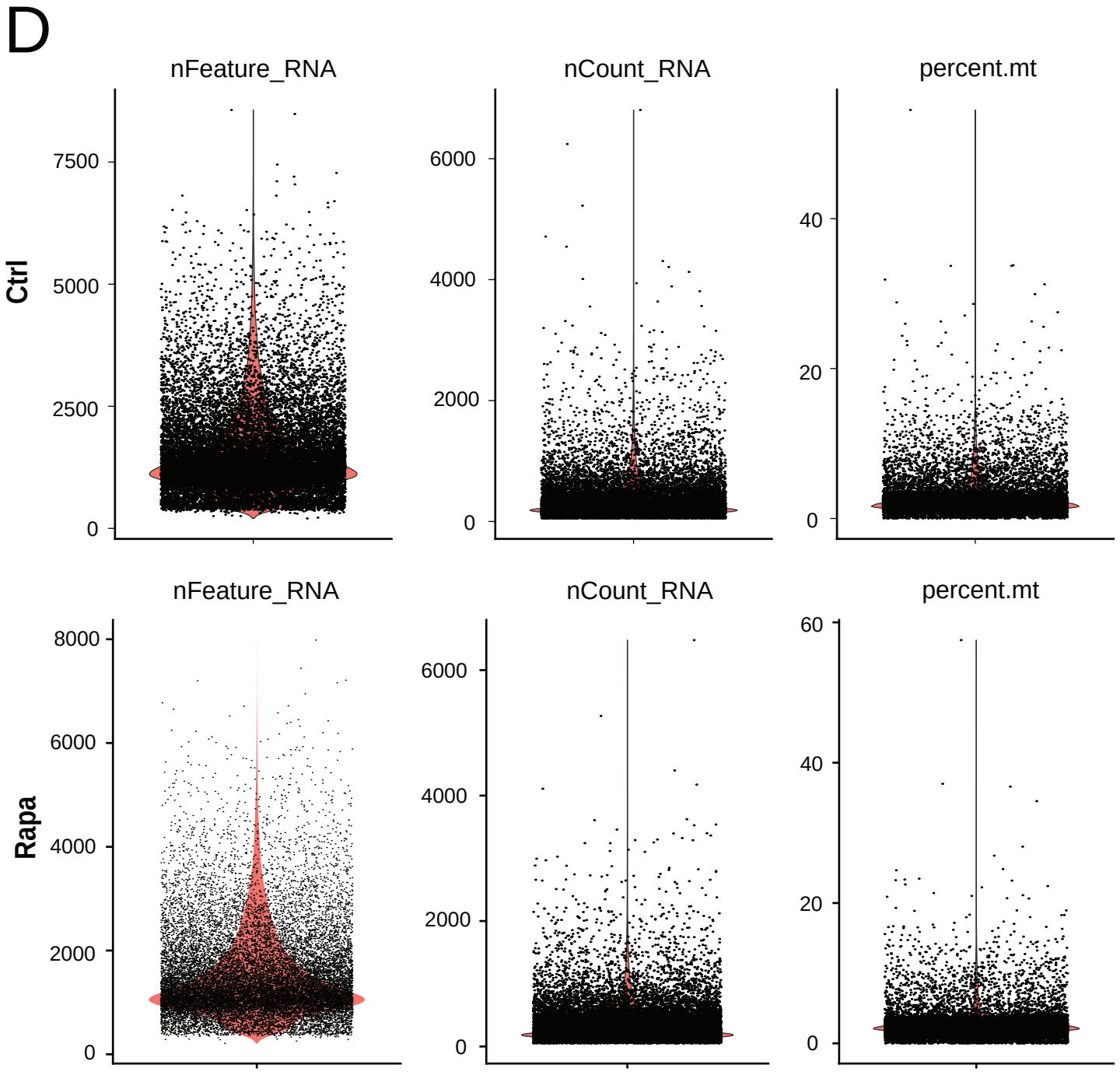

Supplementary Figure 1
