# Supplementary material for: Therapeutic Immune Reprogramming by Rapamycin Attenuates Plaque Inflammation and Lymphoid Immune Responses in Aged Atherosclerotic Mice
Source: Aging Cell. 2026 Sep 23;25(10):e70730. doi: 10.1111/acel.70730 (PMC13599350; doi:10.1111/acel.70730)
Supplement: Supplementary file 6 [file ACEL-25-e70730-s006.pdf]

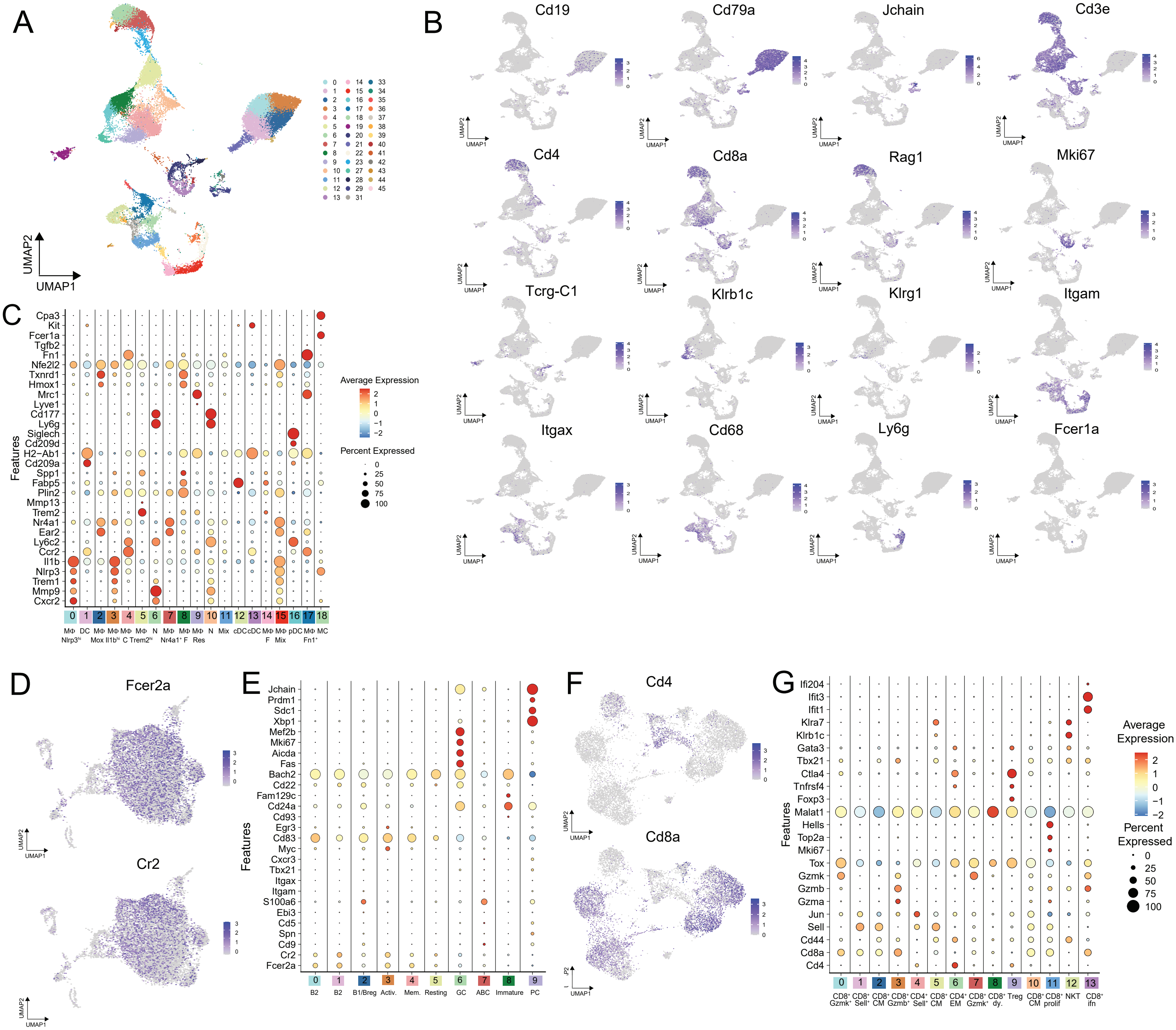

Supplementary Figure 3
